# An Innovative Cloning Platform Enables Large-Scale Production and Maturation of an Oxygen-Tolerant [NiFe]-Hydrogenase from *Cupriavidus necator* in *Escherichia coli*

**DOI:** 10.1371/journal.pone.0068812

**Published:** 2013-07-05

**Authors:** Johannes Schiffels, Olaf Pinkenburg, Maximilian Schelden, El-Hussiny A. A. Aboulnaga, Marcus E. M. Baumann, Thorsten Selmer

**Affiliations:** 1 Department of Chemistry and Biotechnology, Aachen University of Applied Sciences, Juelich, Germany; 2 Institute for Immunology, Biomedical Research Centre (BMFZ), Philipps University of Marburg, Marburg (Lahn), Germany; Louisiana State University and A & M College, United States of America

## Abstract

Expression of multiple heterologous genes in a dedicated host is a prerequisite for approaches in synthetic biology, spanning from the production of recombinant multiprotein complexes to the transfer of tailor-made metabolic pathways. Such attempts are often exacerbated, due in most cases to a lack of proper directional, robust and readily accessible genetic tools. Here, we introduce an innovative system for cloning and expression of multiple genes in *Escherichia coli* BL21 (DE3). Using the novel methodology, genes are equipped with individual promoters and terminators and subsequently assembled. The resulting multiple gene cassettes may either be placed in one vector or alternatively distributed among a set of compatible plasmids. We demonstrate the effectiveness of the developed tool by production and maturation of the NAD^+^reducing soluble [NiFe]-hydrogenase (SH) from *Cupriavidus necator* H16 (formerly *Ralstonia eutropha* H16) in *E. coli* BL21Star™ (DE3). The SH (encoded in *hoxFUYHI*) was successfully matured by co-expression of a dedicated set of auxiliary genes, comprising seven *hyp* genes (*hypC1D1E1A2B2F2X*) along with *hoxW*, which encodes a specific endopeptidase. Deletion of genes involved in SH maturation reduced maturation efficiency substantially. Further addition of *hoxN1*, encoding a high-affinity nickel permease from *C. necator*, considerably increased maturation efficiency in *E. coli*. Carefully balanced growth conditions enabled hydrogenase production at high cell-densities, scoring mg·(Liter culture)^−1^ yields of purified functional SH. Specific activities of up to 7.2±1.15 U·mg^−1^ were obtained in cell-free extracts, which is in the range of the highest activities ever determined in *C. necator* extracts. The recombinant enzyme was isolated in equal purity and stability as previously achieved with the native form, yielding ultrapure preparations with anaerobic specific activities of up to 230 U·mg^−1^. Owing to the combinatorial power exhibited by the presented cloning platform, the system might represent an important step towards new routes in synthetic biology.

## Introduction

Hydrogenases are ancient, highly complex metalloenzymes which catalyze the conversion of molecular hydrogen (H_2_) into protons and electrons. With an equilibrium constant close to unity, the reaction is reversible in accordance with reactant and product concentrations [1]. Due to thermodynamic constraints, however, most hydrogenases catalyze either the activation or production of H_2_ *in vivo*, giving its host the ability to utilize H_2_ as a source of low potential electrons or alternatively dispense excess reducing equivalents as molecular hydrogen to control the cellular redox balance. All hydrogenases are classified into three convergently evolved groups, based on both sequence similarities and the composition of their metal-containing catalytic centre [2]. The largest and most manifold group is represented by the [NiFe]-hydrogenases, found in archaea and diverse groups of bacteria [3]. With regard to their properties and sensitivity towards oxygen, they may be classified in accordance to the environment their hosts were exposed to throughout evolution [4]. Consequently, the most superior enzymes in this regard are found in strictly respiratory organisms living in the presence of O_2_, H_2_ and CO_2_ (‘Knallgas bacteria’). A small group of [NiFe]-hydrogenases are termed ‘oxygen tolerant’, since their catalytic cycle proceeds even under air, which makes these catalysts important targets for the biotechnological industry [5]–[7].

One desirable goal is the implementation of hydrogenases into light-driven routes for biohydrogen production. While the most efficient H_2_ evolving enzymes are [FeFe]- rather than [NiFe]-hydrogenases, their applicability in biophotolysis is hampered by the fact that they suffer irreversible damage upon exposure to oxygen and sunlight [8], [9]. The coupling of both [FeFe]-, and [NiFe]-hydrogenases to the oxygen-independent photosystem I (PSI) has been demonstrated [10], [11]. However, tailored light-driven routes from H_2_O to H_2_, which involve the O_2_-generating photosystem II (PSII), will essentially rely on oxygen-tolerant [NiFe]-hydrogenases.

Apart from H_2_ production, application of these enzymes for utilization/activation of hydrogen is an equally interesting topic, i. e. when hydrogen gas is used to energize processes and catalyses. An oxygen-tolerant hydrogenase was successfully coupled with fungal laccase in a membrane-free enzyme-based fuel cell operating at low hydrogen concentrations [12]. Furthermore, the so-called bidirectional hydrogenases (Class 3d [NiFe]-hydrogenases, [2]) couple hydrogen activation to the reduction of soluble cofactors like NAD(P)^+^, which opens up novel routes for efficient and clean redox balancing *in vitro* [13], [14].

The “Knallgas bacterium” *Cupriavidus necator* (*Cn*, formerly *Ralstonia eutropha* H16) possesses at least three oxygen-tolerant [NiFe]-hydrogenases [15]: The membrane-bound hydrogenase (MBH) consists of three subunits and couples hydrogen uptake in the periplasm directly to the respiratory chain. A cytoplasmic bidirectional hydrogenase (soluble hydrogenase, SH), the target of this project, links H_2_ oxidation to the reduction of NAD^+^, thereby forming reducing equivalents for respiration and CO_2_ fixation. The SH consists of six subunits and can be subdivided structurally into two distinct modules: A hydrogenase moiety HoxYH, accompanied by an NADH:oxidoreductase (diaphorase) module, termed HoxFU, with sequence similarities to the peripheral subunits of mitochondrial Complex I [16]. The hexameric structure is completed by a homodimer of HoxI, a small subunit bound to the diaphorase module, which serves as an NADPH activation site of the oxidized enzyme [17]. Transcriptional regulation of the genes encoding the two known metabolic hydrogenases (SH and MBH) in *Cn* is mediated by a third, strictly regulatory hydrogenase (RH).

The three *Cn* hydrogenases are characterized by superior oxygen tolerance, which in each case is believed to be provided by different mechanisms: In case of the MBH, an unusual FeS cluster proximal to the catalytic core serves as an electron switch with the ability to deliver electrons for the reduction of oxygen to water [18]. The RH shields the [NiFe]-site from oxygen by means of hydrophobic gas channels, which provide steric hurdles for the O_2_ molecule to enter [19]. For the SH, the mode of oxygen tolerance is subject of ongoing discussions. It was initially believed that the SH active site contains two additional cyanide ligands, preventing oxygen species from binding tightly to the bimetallic core [20]–[22]. Since recent studies do not support this hypothesis [23], [24], this topic remains unclear. However, an important role in this mechanism is attributed to the HoxY-bound selectively releasable cofactor FMN-a [24], [25].

**Figure 1:**
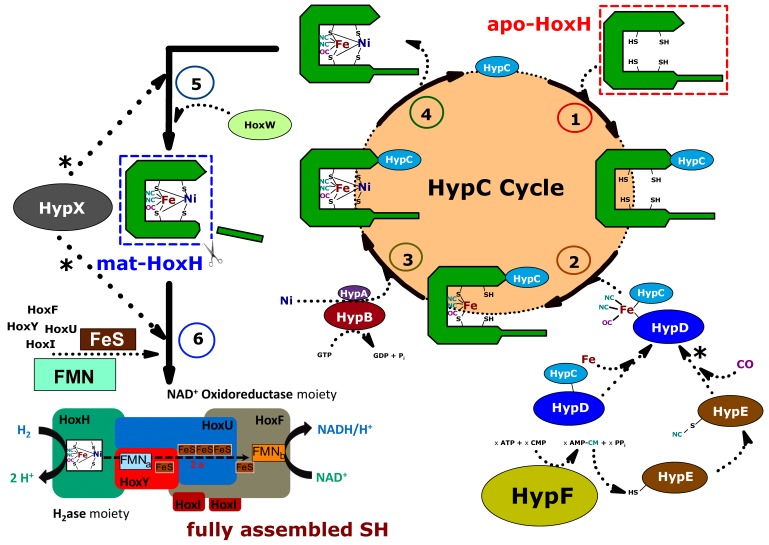
Current model of SH maturation in *Cupriavidus necator*. Steps: **1.** HypC binds to HoxH, preventing improper folding prior metal-center assembly [70], [71]; **2.** Delivery of the iron center comprising the diatomic ligands: The cyanide ligands are derived from carbamoyl phosphate (CMP) in a transcarbamoylation/dehydration reaction catalyzed by HypF/HypE, thereby transferring the carbamoyl moiety to a C-terminal cysteine of HypE [72]–[74]. Modified HypE forms a complex with the preassembled HypCD complex, which is likely to “store” the iron complex until the ligand coordination is completed and subsequently delivers it to apo-HoxH. This step probably involves a redox reaction [75], [76]; **3.** Nickel insertion is mediated by the concerted action of HypA and HypB in a GTP dependent reaction [77]–[79]; **4.** HypC detaches from HoxH; **5.** HoxW cleaves a 24 amino acid peptide off the C-terminus of the HoxH apoprotein. HoxW activity requires pre-incorporated nickel [80], [81]; **6.** HoxH folds and thereby buries the bimetallic core inside the protein (at the hydrophobic contact surface to HoxY). The SH subunits assemble; Prior to this step, FMN cofactors and the iron-sulfur clusters are delivered by the cellular machineries. Unresolved reactions, which include the action of HypX and the origin of the carbonyl ligand are indicated (*).

[NiFe]-hydrogenases are characterized by a highly complex maturation process, in which the catalytic centre is assembled by the concerted action of a set of specific auxiliary proteins (Fig. 1). In this regard, *Cn* SH and MBH are the best studied [NiFe]-hydrogenases aside from the *E. coli* analogues due to the immense biotechnological interest arising from their unique oxygen stability. Some coherencies, however, remain unresolved and are therefore derived from detailed studies on *E. coli* hydrogenase 3 (HycE; reviewed in [26]). It is generally accepted that insertion of the bimetallic core, accompanied by one carbonyl and two cyanide iron ligands, requires the concerted action of *hypABCDEF* gene products (Fig. 1). In addition, a specific endopeptidase, which cleaves a small peptide off the C-terminal domain of the apo-subunit and thus allows formation of the oligomeric enzyme, is essential and specific for each hydrogenase. HypX, an additional maturase, is involved in the maturation of hydrogenases solely found in organisms living in oxic environments and has been proven to be crucial for oxygen-tolerance of these enzymes [20], [27], [28].

In this study, we transferred the capability for production and functional maturation of the SH from *Cn* to *Escherichia coli* BL21Star™ (DE3). In the past, comparable attempts to produce recombinant [NiFe]-hydrogenases have often met with limited success and were restricted to closely related recipients [29]. This might be attributed to both not fully understood, highly specific maturation machineries and a lack of proper genetic tools meeting the demands for multigene expression. Recently, however, progress has been made in production and maturation of these enzymes. Production routes for functional hydrogenases have been successfully transplanted into *E. coli*, including genes from the archaeon *Pyrococcus furiosus* [30], the marine bacterium *Hydrogenovibrio marinus* [31], the cyanobacterium *Synechocystis* sp. PCC6803 [32] as well as the proteobacteria *Alteromonas macleodii* and *Thiocapsa roseopersicina* [33]. Although these recent advances might be considered important steps in the field of applied biohydrogen research, major issues still need to be overcome towards universally applicable large-scale production platforms for these important catalysts.

**Figure 2:**
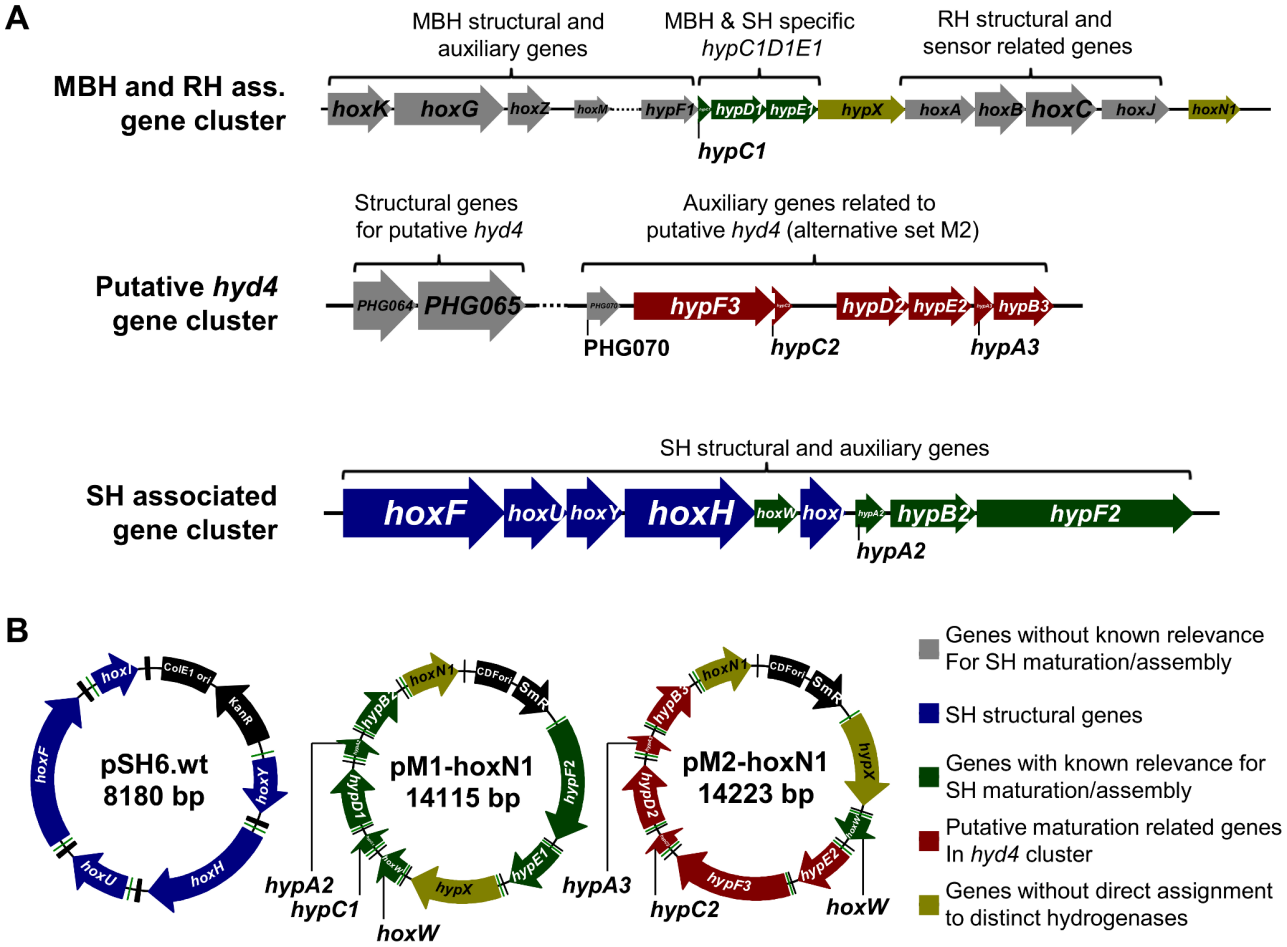
Gene selection for heterologous SH expression in *Escherichia coli*. **a**) Distribution of hydrogenase related genes and putative SH related genes on the pHG1 megaplasmid of *Cupriavidus necator* (*Cn*). pHG1 comprises three distinct hydrogenase clusters (locus MBH cluster: 100–22390; locus *hyd4* cluster: 59962–74032; locus SH cluster: 79712–89227) [52]. The MBH cluster contains the MBH and RH structural genes along with numerous accessory genes for MBH, RH and SH maturation. A partially duplicated set of maturation related genes is present in vicinity to the SH structural genes. The putative structural and auxiliary genes localized in the *hyd4* cluster have not been characterized to date. The *hyp* genes present in this cluster have been tested as an alternative set for SH maturation in this study. Thicker arrows indicate structural genes. **b**) Main expression constructs designed in this study for recombinant SH production in *E. coli*. Plasmids used for maturation and SH production trials were derivatives of the three depicted constructs. The pM1 and pM2 derivatives differ in the origin of the *hypABCDEF* genes (see Table 1). Two independent genes *hypX* and *hoxN1*, as well as the HoxH specific endopeptidase gene *hoxW*, were included in both pM1 and pM2 derivatives. All genes were placed under control of individual T7-promoters and -terminators.

Therefore, in this study, we addressed the major requirements for a multigene expression system, aiming at the ability to either reliably produce such enzymes at high yields, or implement their production circuit into a primary metabolic route, e. g. in synthetic biology approaches. The *Cn* SH, a prototype of oxygen-tolerant [NiFe]-hydrogenases which nicely spans the previously outlined fields of industrial application [5], [14], [34], was chosen as the target enzyme in this study. Production of the SH requires five structural (*hoxFUYHI*) as well as eight auxiliary genes (*hypC1D1E1A2B2F2X* and *hoxW* encoding the specific endopeptidase which finalizes the maturation process) (Fig. 2a). Another gene, *hoxN1*, which encodes a high-affinity nickel-transporter from *Cn*, was included later on as the ninth auxiliary gene. In addition, an alternative set of *Cn* Hyp proteins encoded in the hitherto uncharacterized *hyd4* gene cluster (*hypC2D2E2A3B3F3*) present in the *Cn* genome (Fig. 2a), was tested for complementation of the dedicated set in this study.

We designed a novel cloning and expression platform, which combines the features of three previous studies: The basis of the system was previously developed in our lab [35] and is commercially available as the StarGate® cloning system (IBA, Germany). The system was enlarged by a novel set of fusion vectors, which place each target gene under control of an individual T7 promoter and –terminator. Furthermore, a set of combinatorial acceptor vectors, redesigned from the Duet plasmids (Novagen), was synthesized to fit the functional requirements of the abovementioned system and allow co-expression of an arbitrary number of genes. In order to apply the novel system to the recombinant SH synthesis route, a cultivation/expression strategy based on a T7-autoinduction system [36] was designed to render each gene product soluble and to enable *in vivo* maturation of the SH. This might represent an important technique for studies facing similar challenges in the future. Using the developed platform, we present here the first successful high-yield production of a recombinant oxygen-tolerant [NiFe]-hydrogenase, the SH from *Cupriavidus necator*.

## Results

### Design of the Cloning and Expression System

The cloning system presented in this paper is based on a methodology previously developed in our lab, which utilizes type IIS restriction-ligation (alternating between *Lgu*I and *Esp*3I-mediated excision of the insert and directed transfer to an acceptor vector; Figure S1) [35]. This system is currently marketed as the StarGate® cloning technology (IBA, Germany). A gene fusion strategy was initially implemented in this system, which enables assembly of multiple genes to a polycistronic operon (Figure S2). While this represents a very common strategy in terms of gene assembly, for the *Cn* genes we faced difficulties applying this procedure, since in most cases translation was aborted following the first gene in order (data not shown). We therefore designed a screening strategy (described in the methods section) to establish a functioning order of assembled genes. This was successful for the SH genes (final order: *hoxIYHUF*), but failed for both sets of auxiliary genes.

Since individual production of every *Cn* gene product was demonstrated (data not shown), an alternative fusion system was developed to circumvent the polycistronic assembly step. A novel set of fusion vectors, the pFxT7-series (Figure S3a,b), was designed. The vectors were equipped with the same recognition sites like the “standard” fusion vectors. However, instead of connecting the fused genes via ribosomal binding sites (rbs), the novel vectors equip each gene with an individual T7-promoter and –terminator. As a consequence, the repetitive fusion process using these vectors yields plasmids with multiple genes under individual promoter control (Fig. 2b). As an advantageous secondary action, subcloning of the assembled products into dedicated expression vectors was superfluous, since each intermediate plasmid served as an inducible expression construct.

No maintenance or stability issues were faced with ten or more gene cassettes in one construct. However, expression of both structural and accessory genes from a single plasmid proved disadvantageous, since determination of essential maturation related genes was largely facilitated when the gene sets were separated *a priori* (see deletion experiments below). Two compatible plasmids pSm.CDF.3a and pAmp.RSF.3a (Fig. S3c) were, therefore, redesigned from the Duet™ vectors (Novagen) to match the basic cloning system and allow combinatorial gene expression in concert with pEntry (Kan^R^, ColE1 ori) constructs.

Gene cassettes were assembled systematically, yielding three main assembly modules termed SH (structural genes *hoxFUYHI*), M1 (*hoxW*, *hypA2B2C1D1E1F2X*) and M2 (*hoxW*, *hypA3B3C2D2E2F3X*) (Table 1 and Fig. 2b). For each module, different variants were designed: in case of SH modules, the *hoxI* gene was either provided or omitted, yielding four-subunit (HoxFUYH) or six-subunit (HoxFUYHI_2_) versions of the SH [17]. Both versions were provided as wildtype- and N-terminally StrepII-tagged enzyme variants with the affinity-tag being fused to either *hoxF* (four-subunit SH) or *hoxI* (six-subunit SH). For M1, which represents the dedicated SH maturation set [37]–[39], M1Δ*hypA2B2*, M1Δ*hypC1D1*, M1Δ*hypE1F2*, M1Δ*hypX* and M1Δ*hoxW* were assembled as a means to study the effect of these deletions on SH maturation *in vivo* (see deletion experiments below). M1-*hoxN1* and M2-*hoxN1* contained an additional gene encoding a high-affinity nickel permease HoxN1 from *Cn* in addition to the full sets of M1/M2 genes. Primers and oligonucleotides used in this study are listed in Table S1. A list of basic strains and plasmids is given in Table S2, while SH production and deletion strains are listed in Tables 2 and S3.

**Table 1:** List of *Cupriavidus necator* genes cloned in this project.

Subunit classification/function of putative gene product	SH structural		M1 analogs		M2 analogs		Independent	
	Locus tag	Gene	Locus tag	Gene	Locus tag	Gene	Locus tag	Gene
Diaphorase large subunit	PHG088	*hoxF*						
Diaphorase small subunit	PHG089	*hoxU*						
H_2_ase small subunit	PHG090	*hoxY*						
H_2_ase large subunit	PHG091	*hoxH*						
Diaphorase-associated dimer with NADPH activation site	PHG093	*hoxI*						
Nickel storage and delivery			PHG094	*hypA2*	PHG077	*hypA3*		
Nickel insertion into HoxH with HypA			PHG095	*hypB2*	PHG078	*hypB3*		
HoxH-chaperone; insertion of Fe-center with HypD			PHG015	*hypC1*	PHG073	*hypC2*		
Insertion of Fe-center with HypC; FeS (redox-active protein)			PHG016	*hypD1*	PHG075	*hypD2*		
CN^–^delivery to HypCD after modification by HypF			PHG017	*hypE1*	PHG076	*hypE2*		
CN^–^synthesis from CMP and delivery to HypE			PHG096	*hypF2*	PHG072	*hypF3*		
Specific C-terminal truncation of H_2_ase large subunit			PHG092	*hoxW*	PHG070^a^	*–*		
Uncharacterized; confers oxygen-stability to H_2_ase							PHG018	*hypX*
High-affinity membrane-bound nickel-permease							PHG023	*hoxN1*

a The *hyd4* putative specific C-terminal endopeptidase encoded in PHG070 was not included in the regular pM2 constructs and its derivatives, since HoxW was in different studies proven to be the HoxH specific protease essential for catalytic activity.

CMP = Carbamoyl phosphate.

**Table 2:** Selection of strains, which were generated in this study and used for production of recombinant SH variants.

SH variant^a^	Strain name^b^	Plasmids	Heterologous genes	Specific activity [U·mg^−1^]^c^
wt	K1A	pSH4.wt+pM1	*hoxYHUF//hypF2, E1, X; hoxW; hypC1, D1, A2, B2*	3.2±0.384
	K1A-HoxN1	pSH4.wt+pM1-*hoxN1*	*hoxYHUF//hypF2, E1, X; hoxW; hypC1, D1, A2, B2; hoxN1*	7.2±1.152
1	SH1F	pSH4.Strep+pM1	*hoxYHUF* (5′-StrepII)*//hypF2, E1, X; hoxW; hypC1, D1, A2, B2*	2.2±0.242
	SH1F-HoxN1	pSH4.Strep+pM1-*hoxN1*	*hoxYHUF* (5′-StrepII)*//hypF2, E1, X; hoxW; hypC1, D1, A2, B2; hoxN1*	6.8±1.17
	SHdec1	pASGwt-*hoxUYH*+pM1-*hoxF*.Strep	*hoxYHU//hoxF* (5′-StrepII)*; hypF2, E1, X; hoxW; hypC1, D1, A2, B2*	3.0±0.558
	SHdec3	pASGwt-*hoxYH*+pM1-*hoxUF*.Strep	*hoxYH//hoxU, F* (5′-StrepII)*; hypF2, E1, X; hoxW; hypC1, D1, A2, B2*	1.3±0.122
	NAES4.1	pAmp.RSF.SH4.Strep+pE.M1+pM2	*hoxYHUF* (5′-StrepII)*//hypF2, E1, X; hoxW; hypC1, D1, A2, B2//hypX; hoxW; hypE2, F3, C2, D2, A3, B3*	0.77±0.083
2	SH2F	pSH6.Strep+pM1	*YHI*(5′-StrepII)*UF//hypF2, E1, X; hoxW; hypC1, D1, A2, B2*	1.7±0.18
	SH2F-HoxN1	pSH6.Strep+pM1-*hoxN1*	*YHI*(5′-StrepII)*UF//hypF2, E1, X; hoxW; hypC1, D1, A2, B2; hoxN1*	3.8±0.346

a SH variants: wt = wildtype SH, untagged; 1 = SH variant 1: four-subunit enzyme HoxFUYH with N-terminally StrepII-tagged HoxF; 2 = SH variant 2: six-subunit enzyme HoxFUYHI_2_ with N-terminally StrepII-tagged HoxI.

b All recombinant strains were generated with *E. coli* BL21Star™ (DE3) as the basic strain.

c Specific activities were determined in extracts from cells obtained in three independent ‘autoinduction’ batches under optimized growth conditions outlined in the methods section of the paper. Given values are arithmetic means of the triplicate measurements. Statistical values indicated (±) represent standard deviations. 1 Unit is defined as the H_2_-mediated reduction of 1 µmol NAD^+^per minute.

### Production and Purification of Recombinant SH

*E. coli* BL21Star™ (DE3) (Invitrogen) served as the basic strain for SH production trials. Expression strains were readily generated by sequential transformation with the respective plasmid constructs. Since each compatible plasmid (pEntry, pSm.CDF.3a and pAmp.RSF.3a) carries a unique origin of replication and thus, provides a different copy number per cell, co-expression was tested using variable vector combinations. As outlined below, strains carrying SH genes on pEntry (termed pSH-derivatives) in combination with M1 genes on pSm.CDF.3a (termed pM1-derivatives) served as the most efficient SH production strains (K1A-, K1B-, SH1F- and SH2F-strains; Table 2). NAES strains harbored SH genes on a third, high-copy plasmid pAmp.RSF.3a, while auxiliary genes (M1 or M1 & M2) were distributed among pEntry and pSm.CDF.3a (Table 2). A third combination of plasmids was utilized using pASG-derivatives (carrying polycistronic SH-modules) with pSm.CDF.3a-constructs. These strains (SHdec strains; Table 2) were used to study decoupled induction of SH (tetracycline regulon) and M1 (lacUV5/T7 RNAP promoter) gene expression (see below).

**Figure 3:**
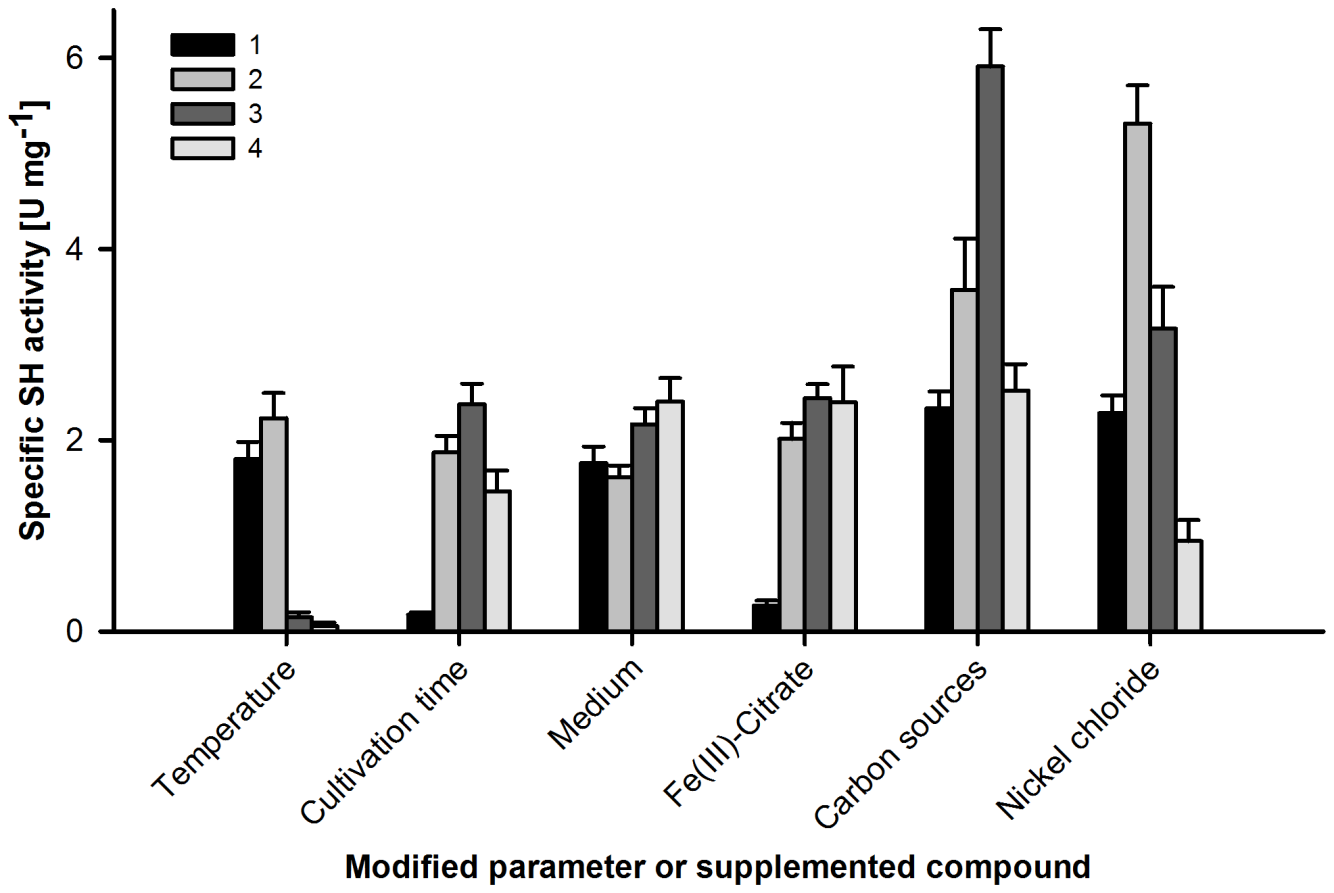
Selection of Optimization Trials for Maximized SH Production in Recombinant Strains. Specific activities were determined in extracts of cells from three independent cultivation trials. Error bars indicated represent standard deviations. Parameters were modified as follows: *Temperature* (**1**∶18°C; **2**∶25°C; **3**∶30°C; **4**∶37°C), *Time* (**1**∶24 h; **2**∶30 h; **3**∶36 h; **4**∶42 h), *Medium* (**1**: LB; **2**: TB; **3**: HEM; **4**: M9), *Ferric ammonium citrate* (**1**∶0 µM; **2**∶50 µM; **3**∶100 µM; **4**∶500 µM), *C-sources* (given as % (wt/vol) glucose/% (vol/vol) glycerol/% (wt/vol) lactose; **1**∶0.05/1/0.2; **2**∶0.1/2/0.4; **3**∶0.1/2/0.8; **4**∶0.2/2/0.8), *NiCl_2_* (**1**∶0 µM; **2**∶1 µM; **3**∶25 µM; **4**∶100 µM).

Production of functional SH was initially tested by inducing gene expression with Isopropyl-β-D-thiogalactopyranoside (IPTG) under aerobic as well as anaerobic growth conditions and varying temperatures. Although the gene products were detectable in whole-cell lysates by SDS-PAGE (data not shown), SH activity was lacking. An alternative T7-promoter based approach, which employs growth-phase dependent lactose-triggered induction of T7 RNAP gene expression following glucose consumption, was subsequently tested. This strategy was originally introduced by Studier [36] and termed ‘autoinduction’. This proved to be a flexible approach, since growth and expression parameters were widely controllable by modification of both media formulations and physical conditions (Fig. 3).

Prolonged cultivation using autoinduction in semi-defined media enabled functional production and maturation of SH variants in recombinant strains. In the course of initial experiments it was found that in *E. coli*, functional maturation of the SH relies on low-temperature (22–25°C) cultivation. For maximized yield, cells were harvested in the late stationary phase (36–40 hours after inoculation; Fig. 3). Initial activities in cell-free extracts (strains K1A, SH1F, SH2F; Table 2) were in the range of 0.1–0.6 U·mg^−1^ and suffered from poor reproducibility. Since efficient endogenous nickel uptake systems in *E. coli* are FNR dependent and require anaerobiosis [40], we introduced the *hoxN1* gene encoding a high-affinity nickel permease from *Cn* [41]–[43] (plasmids pM1-*hoxN1* and pM2-*hoxN1*, Fig. 2b), enabling nickel uptake under aerobic conditions. Indeed, co-expression of *hoxN1* increased maturation efficiency by a factor of about 2–3 (Fig. S4). In search for a balanced expression strategy in consideration of high-level production, minimization of inclusion body formation, functionality of the maturation machinery as well as stability of the matured SH, a series of optimization trials was carried out with sequential modifications of media formulations and cultivation parameters (Fig. 3). K1A-HoxN1 (pSH4.wt and pM1-*hoxN1*) served as the test strain in these experiments.

SH production was favored using M9 minimal medium. Supplementation of both iron (added as ferric ammonium citrate) and nickel were crucial for efficient SH maturation in this system (Fig. 3). However, 1 µM NiCl_2_ was sufficient in all expression strains. Elevated levels led to decreased SH activity except for K1A ΔHypA2B2 (Fig. S4). In strains with HoxN1, concentrations above 1 µM substantially reduced SH activity and even diminished cell growth, a behavior which might be attributable to intracellular accumulation of Ni^2+^up to toxic levels. A similar behavior was observed for *Cn* under heterotrophic growth conditions [44]. Beside nickel and iron, supplementation of riboflavin (1 µM) as a precursor of FMN proved to increase SH activity. The addition of an amino acid mixture (1 mM each) was tested and proved to increase SH activity by a factor of about 1.3 (data not shown). However, the same effect was achieved by supplementation of 5% (v/v) LB-medium, which henceforward served as the cheaper alternative. Supplementation of cysteine (1 mM) did not further increase activity (data not shown). The state of the preparatory culture at the time of inoculation proved to have an important effect on the quality of the cells. Details about this step are given in the methods section of the paper. Using optimized media in combination with the determined growth parameters yielded the SH activities listed in Table 2.

Cytoplasmic specific activities were in the range of 1–3.5 U·mg^−1^ for strains carrying pSH and pM1 plasmids (strains K1A, SH1F, SH2F; Table 2). Strains carrying the *hoxN1* gene yielded specific activities up to 7.2 U·mg^−1^, which is in line with the highest SH activities ever observed in *Cn* extracts [45]. Strategies involving growth-phase decoupled expression of the SH and auxiliary genes were tested by combination of Tet- and T7-promoter controlled genes (SHdec strains; Table 2). The idea behind the generation of SHdec strains was mainly to allow synthesis of the SH subunits in a cellular environment where the maturation machinery was already present, thereby preventing aggregation of the unassembled polypeptides. However, the highest activities obtained with these strains were in the same range of those from K1A, SH1F and SH2F (Table 2).

### Properties of Recombinant and Native SH

**Figure 4:**
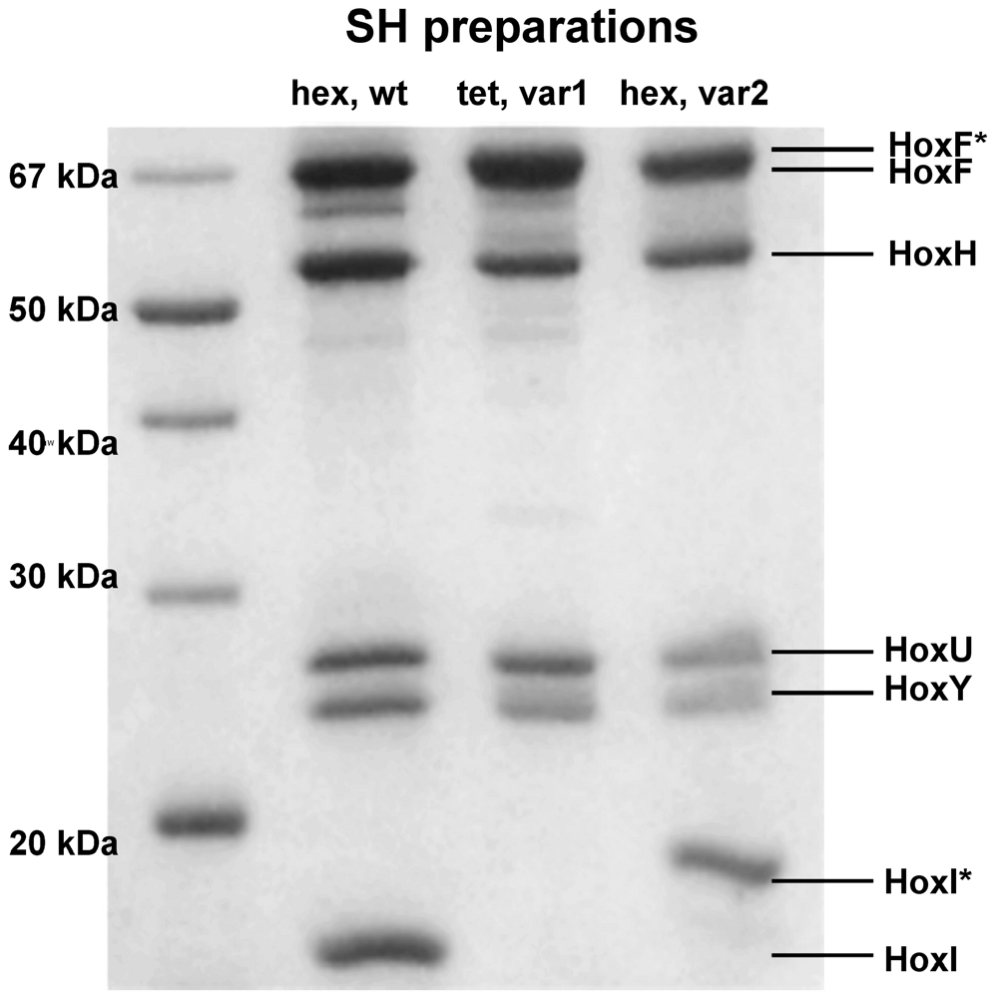
SDS-PAGE Analysis of Enzyme Preparations. 5 µg of protein were applied to each lane and separated on a 12% SDS-gel; Legend: SH_hex,wt_ = wildtype six-subunit SH purified from *Cn*; SH_tet,var1_ = four-subunit SH purified from recombinant strain SH1F (N-terminally-StrepII tagged HoxF; HoxF*); SH_hex,var2_ = six-subunit SH purified from recombinant strain SH2F (N-terminally-StrepII tagged HoxI; HoxI*).

Correct assembly and functionality of the recombinant SH was further demonstrated by purification of recombinant SH, using strains SH1F (four-subunit SH, N-terminally StrepII-tagged HoxF) and SH2F (six-subunit SH, N-terminally StrepII-tagged HoxI; Table 2) for production. Both preparations were homogenous according to SDS-PAGE analysis (Fig. 4) and exhibited anaerobic H_2_:NAD^+^activities of 192 and 227 U·mg^−1^, respectively, displaying the highest anaerobic activities ever obtained for the SH (Tables 3 and 4) [45]. Native six-subunit SH used for comparison was purified from *Cn* as described in [45] to a specific activity of 130 U·mg^−1^ (Table 5). Accurate assembly of the SH catalytic core is readily evidenced by measuring the enzyme’s physiological activity (H_2_:NAD^+^), since electron flow from the [NiFe] active site via FMN-a to FMN-b over an array of FeS clusters was shown to be a prerequisite for this assay [25].

**Table 3:** Purification table for recombinant SH variant 1 (four-subunit enzyme with 5′-Strep tagged HoxF) purified from recombinant *E. coli* strain SH1F cells.

Fraction	Protein [mg]	Activity [U]^a^	Specific Activity [U·mg^−1^]	Purification fold	Yield
CFE	1,825	1,810	1.0	1	100%
Strep-Tactin Superflow	5	329	67	67	18%
Gel filtration	0.7	125	192	193	7%

About 6 g of wet packed cells were used for purification.

a H_2_:NAD^+^physiological activity, measured under anaerobic conditions. 1 Unit is defined as the H_2_-mediated reduction of 1 µmol NAD^+^per minute.

**Table 4:** Purification table for recombinant SH variant 2 (six-subunit enzyme with 5′-Strep tagged HoxI), purified from recombinant *E. coli* strain SH2F cells.

Fraction	Protein [mg]	Activity [U]^a^	Specific Activity [U·mg^−1^]	Purification fold	Yield
CFE	2,276	2,754	1.2	1	100%
Strep-Tactin Superflow	45	885	19	16	32%
Gel filtration	2.7	604	227	187	22%

About 7 g of wet packed cells were used for purification.

a H_2_:NAD^+^physiological activity, measured under anaerobic conditions. 1 Unit is defined as the H_2_-mediated reduction of 1 µmol NAD^+^per minute.

**Table 5:** Purification table for endogenous SH purified from *Cupriavidus necator* cells.

Fraction	Protein [mg]	Activity [U]^a^	Specific Activity [U·mg^−1^]	Purification fold	Yield
CFE	1,361	10,903	8.0	1	100%
ASF precipitate	671	8,529	13	1.6	78%
DEAE Sepharose	69	5,828	84	11	53%
Gel filtration	17	2,195	130	16	20%

About 20 g of wet packed cells were used for purification.

a H_2_:NAD^+^physiological activity, measured under anaerobic conditions. 1 Unit is defined as the H_2_-mediated reduction of 1 µmol NAD^+^per minute.

**Figure 5:**
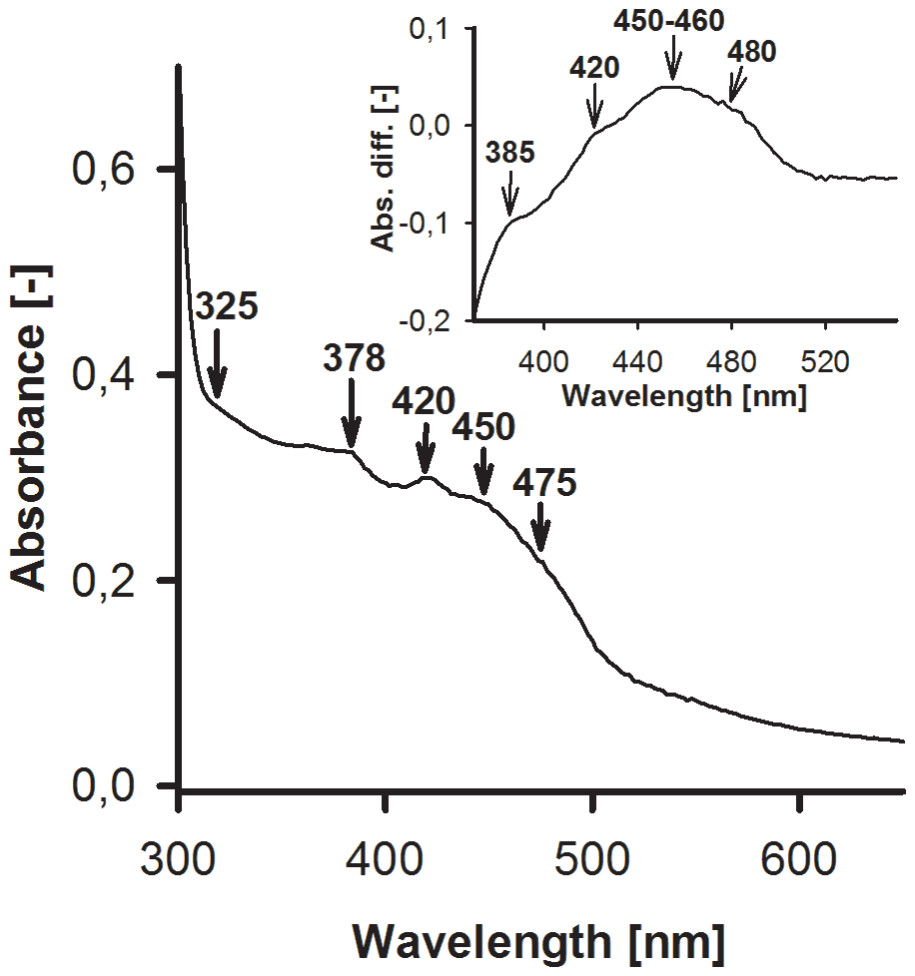
UV/Vis Spectroscopy of Recombinant SH. Main: Spectrum of purified, oxidized SH_var2_ (1 mg·mL^−1^); Inset: Difference spectrum between oxidized and dithionite (500 µM) reduced enzyme.

For purification of both native and recombinant SH, controlled redox conditions prior to the first chromatographic step were highly beneficial for the final quality of enzyme preparations (addition of 50 mM succinate and keeping the suspension under argon during cell opening and centrifugation; Table S4). Although the isolated SH is most stable in its fully oxidized form, this phenomenon has been observed in a previously published work, where the most active SH preparations to date were obtained by applying these conditions [45]. Iron contents of pure enzymes were determined colorimetrically. We obtained values of 15–17, 15–18 and 16–19 Fe atoms per hydrogenase complex for the purified SH_var1_, SH_var2_ and the native SH, respectively, which is slightly below the 19 iron atoms predicted but appears to be consistent in all variants (Table S5). The presence of both [2Fe2S] and [4Fe4S] clusters was further observed by UV/Vis spectroscopy, displaying prominent peaks of the oxidized enzyme at 325 and 378 nm as well as 420 and 475 nm, respectively [46]–[48]. Difference spectra provided further evidence for the presence of flavins (FMN), which contribute to the absorption peaks at 370–390 as well as at 450 nm [49], [50] (Fig. 5).

Stabilities of recombinant SH variants under air as well as inactivation under anaerobic conditions (2–5% H_2_, rest N_2_) resembled those of the native SH (Table S5) [51]. In untreated cell-free extracts prior purification, the recombinant variants were comparably labile. This was rather attributed to the redox conditions in *E. coli* extracts than to proteolytic activity, since treatment with succinate under an argon atmosphere stabilized the enzyme (Tables S4, S5). The four-subunit and six-subunit recombinant SH variants had molecular masses of ∼171 and 213 kDa, respectively, as determined by gel filtration (Fig. S5). Activation of the oxidized enzyme variants was readily achieved by addition of NADH (5 µM; both variants) or NADPH (40 µM; only six-subunit variant SH_var2_) to the assay mixture (Table S6). Omission of these activators in assays with purified oxidized SH led to significant lag phases before maximum velocity was reached (Table S6). Both native and recombinant SH were able to catalyze H_2_ consumption in the presence of oxygen. Enzyme activities in such aerobic assays were reduced by 20–25% compared to anaerobic measurements (Tables S5, S6). Taken together, the properties of the recombinant SH variants nicely resembled those of the native enzyme and are in agreement with the data previously published on the four- and six-subunit SH [17], [20], [45], [51].

**Figure 6:**
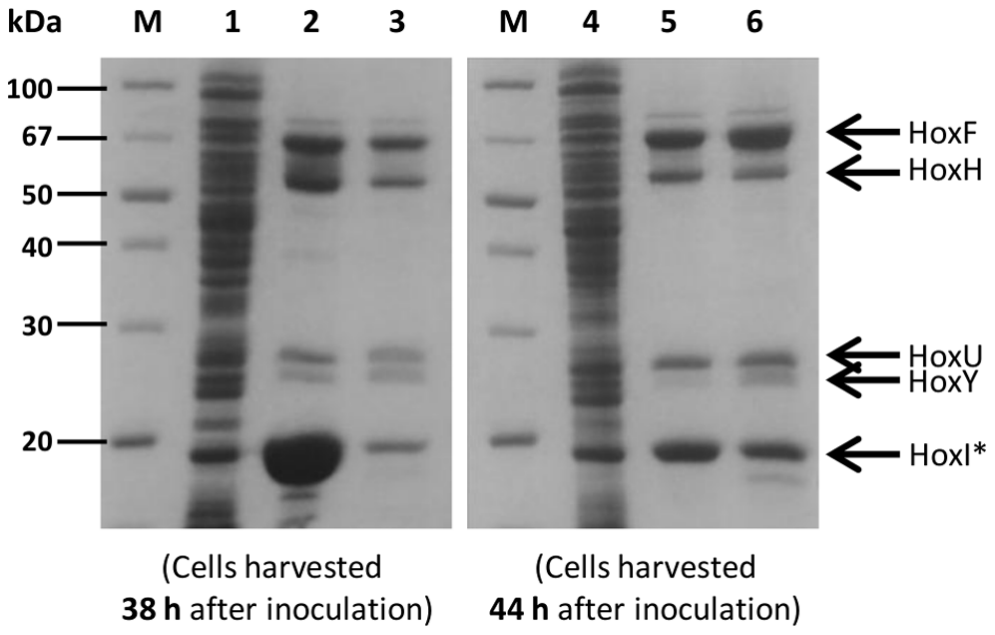
SDS-PAGE analyses of SH preparations from cells, which were harvested after different cultivation times. Cells from strain SH2F (six-subunit SHvar2; 5′-StrepII tagged HoxI; HoxI*), cultivated under ‘optimized autoinduction conditions’, were harvested after 38 hours (lanes 1–3) and 44 hours (lanes 4–6) from the same culture. 1 gram of cells thus obtained was used for preparation of the cell-free extracts (lanes 1 & 4; 25 µg applied). Purification was carried out using a 1 mL StrepTactin Superflow® gravity flow column (lanes 2 & 5; 15 µg and 5 µg applied, respectively) and subsequently a Superdex 200 HR 10/300 gel filtration column for polishing (lanes 3 & 6; 4 µg and 5 µg applied, respectively). Notably, the HoxFUI_2_ diaphorase module appears to be stable over time, while the HoxYH hydrogenase moiety dissociates from the complex after 38–44 hours of cultivation. Activities of the preparations after gel filtration: SH_38 h_: 103 U·mg^−1^; SH_44 h_: 21 U·mg^−1^. M = Protein marker.

As outlined above, the most active recombinant SH preparations were obtained from cells subjected to an ‘optimized growth strategy’ (see methods section). For maximized yield, cells were ideally harvested in semi-anaerobic late stationary phase 36–40 hours after inoculation (Fig. 3). Prolonged cultivation beyond that harvesting window resulted in destabilization and subunit dissociation as observed by purification of detached subunits (Fig. 6). The four-subunit SH variant was less prone to destabilization (data not shown). When the oxidized, fully assembled forms were compared, however, it was apparent that yields were generally higher for the six-subunit variant (Tables 3 and 4), which might be a result of superior tag accessibility in StrepII-HoxI.

### Investigation of Essential Maturation Factors in SH Assembly – a Combinatorial Approach

The development of a robust and efficient cloning methodology enabled comprehensive deletion and complementation experiments, which are usually restricted to knockout trials in native hosts. In this study, we investigated two sets of Hyp proteins encoded in different regions of the *Cn* pHG1 megaplasmid (Fig. 2a and Table 1) for their SH maturation capability. The M2 genes belong to a yet uncharacterized operon (*hyd4*), which harbors two putative [NiFe]-hydrogenase structural genes (PHG064, 65), a complete set of *hyp* genes (*hypF3C2D2E2A3B3*) and a gene PHG070 encoding a putative endopeptidase. A functional overlap in MBH- and SH-specific maturation has previously been observed for parts of the *Cn* Hyp machinery [39]. The SH-associated operon harbors a partial set of duplicated *hyp* genes (*hypA2B2F2*; Fig. 2a) [39], [52]. We included the M2 genes as a means to investigate their capability to replace the M1 genes, since the products of this set have not been characterized previously. Using the combinatorial vectors synthesized in this study, deletion and complementation studies were readily performed by placing the different M1 ‘deletion modules’ in the pSm.CDF.3a-plasmid. Combination of the resulting constructs with pSH4.wt (encoding wildtype four-subunit SH) in *E. coli* BL21Star™ (DE3) enabled functional testing of *in vivo* maturation (Table S3).

**Figure 7:**
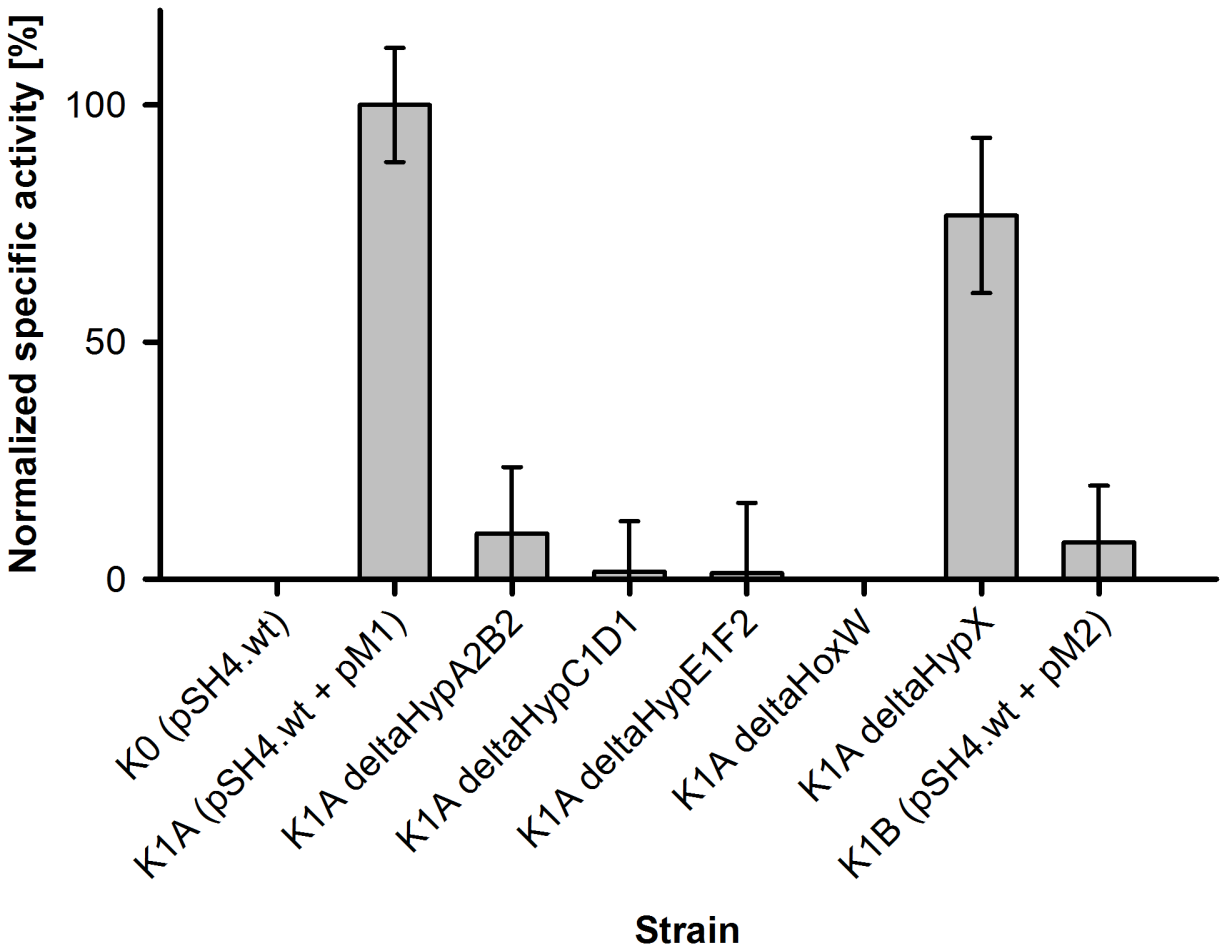
Deletion experiments for *in vivo* maturation of the SH in *Escherichia coli*. Strains and values are listed in Table S3; Control strains: **K0**: pSH4.wt (structural genes without maturation related genes); **K1A**: pSH4.wt and pM1 (structural genes and complete M1 set; 100%; 1.95±0.24 U·mg^−1^). Deleted complexes or proteins omitted are indicated for the K1A deletion strains. **K1B** represents the substitution strain, in which the M1 Hyp proteins are replaced by the M2 analogs (pSH4.wt and pM2). Results are given as specific activities exhibited by extracts obtained from three independent experiments, which were normalized for control strain K1A. Error bars indicated represent standard deviations.

Strain K1A (pSH4.wt+pM1; Table S3) served as the control strain with an SH activity of 1.95±0.24 U·mg^−1^ in cell-free extracts. Except for the negative control strain K0 (SH genes only) as well as K1A ΔHoxW, all extracts from deletion strains exhibited residual SH activity (Fig. 7 and Table S3). Omission of the complexes HypC1D1 and HypE1F2, which are involved in assembly and insertion of the iron center, resulted in substantially reduced activity. The results recorded after deletion of the so far uncharacterized maturase HypX resembled those of previous studies [45], which pointed out minor effects on initial activity (77% activity of K1A) but to a severely altered catalytic behavior in presence of O_2_ (Table S6). Omission of the nickel insertion complex HypA2B2 resulted in moderate effects compared to HypC1D1 and HypE1F2 deletions and could be partially restored by provision of an excess of NiCl_2_ in the medium (Fig. S4). This has previously been demonstrated for the SH in *Cn* [39].

Complete substitution of the six M1 *hyp* genes by their M2 analogs (strain K1B; pSH4.wt+pM2; Table S3) resulted in specific activities one order of magnitude lower (0.15 U·mg^−1^ compared to 1.95 U·mg^−1^ with K1A; Fig. 7 and Table S3), proving that the M2 proteins are less effective in SH maturation than the M1 analogs. However, the activities exhibited by K1B extracts were still significantly higher than those derived from HypC1D1 and HypE1F2 deletion strains (Fig. 7 and Table S3). Hence, the M2 Hyp machinery is active and can, to some degree, complement the M1 analogs. In order to investigate the potential interplay of both M1 and M2 in the course of SH maturation, strain NAES4.1 was designed, which harbored the SH genes on a multicopy plasmid (pAmp.RSF.3a), the M1 module on pEntry and M2 on pSm.CDF.3a. Derivative strains were designed, which carried SH, M1 and M2 modules on the compatible vectors in each possible combination (data not shown). However, maximum activity for the combined SH/M1/M2 co-expression, which was achieved with NAES4.1, amounted to only 0.77 U·mg^−1^ (Table 2).

Supplementation of NiCl_2_ at levels above 1 µM did not increase SH activity in the recombinant strains listed in Table 2. A stimulating effect on maturation was only observed for strain K1A ΔHypA2B2, where deletion was partly complemented by extracellular nickel (Fig. S4). In strains harboring the gene *hoxN1*, activity was substantially increased. This was observed for both K1A-HoxN1 and K1B-HoxN1 (Table 2). However, in these strains, nickel supplementation above 1 µM affected activity adversely (Fig. S4). These findings demonstrate the effectiveness of the heterologous nickel transporter, which at elevated concentrations might accumulate intracellular Ni^2+^to toxic levels.

## Discussion

In light of the continuing progress in omics (genomics and proteomics) and the amount of new data acquired thereby, new grounds in synthetic biology are broken and state-of-the-art genetic tools are becoming limiting technologies. Multigene co-expression systems with high-yield capacity are desirable platforms in this regard, especially when flexible combinatorial approaches are implemented. In this study, we present a large-scale multigene expression platform for *Escherichia coli*, to our knowledge the first of its kind. The basic cloning system uses type IIS restriction endonucleases *Lgu*I and *Esp*3I and was previously designed in our labs [35]. The advantages of this system were already discussed in the corresponding reference and also in a previous work, where an analogous technique was used [53].

Although gene fusion strategies are nowadays implemented in recombinational cloning systems, the drawbacks in the use of artificial polycistrons are nicely demonstrated by the initial problems faced in this study (see above). On the one hand, artificially constructed polycistrons introduce problems related to mRNA secondary structure, stability and ribosomal accessibility. Furthermore, it is known that the expression level of a certain gene placed in a polycistron decreases with increasing distance to the promoter [54], [55]. An alternative approach is the equipment of each gene with an individual promoter and terminator, which has been shown to provide substantially higher amounts of the second gene product in order [55]. Furthermore, due to the translation abortion issues faced for the *Cn* genes, the ‘individual promoter strategy’ became an essential implement. Since high-level expression in *E. coli* often coincides with inclusion body formation, we chose a promoter which enables different induction strategies: The T7 promoter has basal expression-, IPTG induction- or lactose−/‘autoinduction’ [36] capability. As evidenced for SH production and maturation, induction/expression techniques, which allow flexible and extended cultivation strategies, are in some cases inevitable. In view of the SH as a potential NADH regenerating catalyst whose implementation into a tailored metabolic route (like a solvent producing pathway; see below) represents a desirable goal, the aforementioned flexibility required for its functional production is certainly necessary for such combined routes as well.

We further enlarged our system by a set of compatible plasmids and demonstrated the effectiveness of the combined platform. While the use of three different plasmids in one cell was sufficient for co-expression of eighteen genes in this study (strain NAES4.1; Table 2), at least one more compatible vector could be designed from the Duet system (Novagen) on demand. Bearing in mind that at least ten gene cassettes may be placed in one vector (e. g. pM1-*hoxUF*.Strep; Table S2), the combined system is suited for comprehensive metabolic routes comprising at least 40 heterologous genes. As an example, the introduction of an NAD^+^reducing hydrogenase like the SH into metabolically engineered microbes would yield the additional reducing power required for product formation of highly reduced compounds like butan-1-ol *in vivo* [56]. In addition, compatible plasmids may be used as a means to control gene doses of separate modules. In this study, we tested recombinant strains carrying SH structural or auxiliary gene modules on different compatible plasmids (strain list Table 2). The ideal strategy proved to be the use of pEntry and pSm.CDF.3a. Strains employing pAmp.RSF.3a (multicopy) constructs did not yield higher SH activities. However, this might be partly attributed to the poor long-term stability of carbenicillin [57]. The different activities exhibited by the numerous strains are an indicator that gene distribution among plasmids with different origins of replication represents an important parameter for optimization of multigene expression techniques. Furthermore, compatible plasmids are prerequisites for comprehensive deletion or substitution trials as carried out in this study, and might serve as an important tool to replace intricate knockout studies in native hosts.

The SH is a well-studied prototype of bidirectional oxygen-tolerant hydrogenases. Production strategies have been continuously optimized ever since Friedrich and coworkers made the discovery that *Cn* synthesizes its metabolic hydrogenases (MBH and SH) even in the absence of H_2_ under ‘hydrogenase-derepressing’ heterotrophic growth conditions [58]. It has been concluded, that formation of these autotrophic key enzymes is generally favored under cellular conditions of energy limitation, combined with the presence of excess reducing equivalents [59]. In *E. coli*, the stress conditions present in late stationary phase cells represents the ideal environment for activity of the recombinant SH maturation machinery. We further evidenced that nickel transport represents a crucial element for efficient maturation of the SH, which was achieved by co-expression of a high-affinity nickel transporter from *Cn*. Since activity of the endogenous hydrogenases of *E. coli* BL21 (DE3) can be restored by provision of excess extracellular NiCl_2_ [60], a role of the host’s own Hyp analogs in aiding SH maturation cannot be excluded. The cellular conditions during the late stationary phase might be favorable for the functionality of the *E. coli* Hyp proteins, among which at least one (arguably HypD) catalyzes an oxygen-sensitive step [61]. The fact that deletion of SH maturation complexes leads in most cases to severely reduced, but not abolished residual activity (Fig. 7 and Table S3), further strengthens the argument that *E. coli* Hyp proteins might complement the *Cn* analogs to a certain degree. Such interchangeabilities of HypABCDEF proteins have been observed in comparable studies before: The SHI of *Pyrococcus furiosus* can be fully processed by the *E. coli* maturation machinery when only the specific protease FrxA is provided [30]; auxiliary proteins from *Alteromonas macleodii* are able to complement for *Thiocapsa roseopersicina* counterparts [33] and the Hyp proteins from *Cupriavidus necator* were shown to functionally process the SH from *Rhodococcus opacus*, which is, however, highly similar to the *Cn* SH [62]. In this study, we provided an additional example for such interchangeability by replacing the M1 *hyp* genes with the M2 analogs (Fig. 2 and Table 1). The M2 maturases were able to complement the dedicated M1 set to about 8% (Fig. 7 and Table S3).

We demonstrate the first high-yield production platform for a [NiFe]-hydrogenase. Levels of functional recombinant SH in cell-free *E. coli* extracts equaled the highest activities ever obtained with *Cn* [45]. This achievement gives rise to the conclusion that - contrary to previous attempts [30], [32], [33] - our system provides saturating levels of the dedicated maturation proteins. Employing a two-step purification procedure, high-quality recombinant SH preparations were obtained. Specific activities of purified recombinant SH variants were in the same range as the most active preparations ever obtained from *Cn* ([45] and Tables 3,4). From 600 mL ‘autoinduction’ culture, 2.7 mg of SH were routinely purified (Table 4). Given the fact that specific activities in extracts were ultimately improved by a factor of 6 in strains harboring HoxN1 (Table 2), the yields obtainable from those cells are expected to be even higher. A theoretical value of 27 mg ultrapure [NiFe]-hydrogenase per Liter culture might thus be achieved. Therefore, when subjected to carefully balanced growth conditions, *E. coli* represents an ideal host for the production of recombinant oxygen-tolerant [NiFe]-hydrogenases. Since the potential of our SH synthesis system equals or even outmatches the well-established strategies for native SH_wt_ production ([45] and this study), it seems likely that in *Cn*, the endogenous production machinery (both structural and auxiliary proteins) is not used to full capacity. This might be due to energetic constraints, since under heterotrophic growth conditions hydrogenase production is a waste of energy. Activities of the SH in both extracts and pure fractions varied considerably in different reports on the enzyme since its first isolation [17], [20], [45], [51], [58], [63]. The heterologous production platform introduced in this study represents the first reproducible large-scale system for this enzyme to date.

### Conclusions

In this study, we present an innovative solution for the high-yield production of enzyme complexes requiring sophisticated, protein-assisted maturation circuits for functional assembly. The developed platform was successfully exploited for the production of recombinant soluble [NiFe]-hydrogenase (SH) from the ‘Knallgas’ bacterium *Cupriavidus necator* in *Escherichia coli* BL21Star™ (DE3). Owing to the combinatorial power exhibited by the newly developed platform, the system will allow innovative strategies and routes in synthetic biology.

## Materials and Methods

### Molecular Biology Basic Techniques

Primers and oligonucleotides used in this study are listed in Table S1. Cloning and vector assembly was based on one step restriction-ligation reactions using type IIS restriction enzymes *Esp*3I and *Lgu*I along with T4 ligase as recently described [35] (Figs. S1, S2). Unless otherwise stated, PCR amplificates, annealed oligonucleotides and linearized vector backbones were purified using GeneJet PCR Purification Kit (Fermentas), diluted where appropriate and subsequently analyzed using MCE-202 MultiNA chip electrophoresis (Shimadzu).

Newly generated plasmid constructs were transformed into chemically competent *E. coli* DH5α cells via heat shock [64]. Transformants were selected on LB agar plates supplemented with 50 µg·mL^−1^ antibiotics (carbenicillin, kanamycin or spectinomycin) and if necessary 25 µg·mL^−1^ X-Gal, enabling blue/white screen. For pAmp.RSF.3a constructs, the carbenicillin concentration was reduced to 25 µg·mL^−1^. Plasmid purification was performed using GeneJet Plasmid Miniprep Kit (Fermentas). Portions of purified plasmid were subjected to restriction digest using appropriate in-gene cutters (*Nla*III, *Hind*III, *Hinf*I or *Hae*III). The fragmentation pattern was subsequently analyzed using MCE-202 MultiNA chip electrophoresis (Shimadzu). All generated nucleic acid samples were quantified and qualified using NanoDrop ND-1000 spectrophotometer (Peqlab).

### De novo Syntheses of pFxT7 Fusion Vectors

The pFxT7 fusion vectors (Fig. S3a,b) were constructed from the basic vectors pFn1 (upstream fusion) and pFc1 (downstream fusion), which contained a ColE1 origin, an Amp/Carb resistance gene, two convergent *Lgu*I-out sites and two divergent *Esp3*I-in sites, with the latter leaving 4 base overhangs compatible to the ones found in the StarGate® vectors pNFUSE and pCFUSE for fusion. In order to insert the T7-controlled expression cassette with or without N- or C-terminal StrepII-tags, the StarGate® vectors pPSG-wt, pPSG-3 and pPSG-5 were used as templates for amplification of the six cassettes (two amplificates per pPSG derivative for the insertion in pFn1 and pFc1, respectively), using the primers T7cas-pFc-s and T7cas-pFc-as as well as T7cas-pFn-s and T7cas-pFn-as. Residual vector template was cut with *Dpn*I and the T7 cassettes were inserted into pFn1 or pFc1 using *Lgu*I and T4-ligase in one pot reactions. Since *lacP/Z* from pPSG derivatives was intentionally carried over, the newly designed fusion vectors pFnT7(wt), pFnT7(3′), pFnT7(5′), pFcT7(wt), pFcT7(3′) and pFcT7(5′) adopted this feature, enabling blue/white screen capability as known from the hitherto existing StarGate® fusion system.

### De Novo Syntheses of Compatible Expression Constructs Based on Duet™ (Novagen)

The vectors pAmp.RSF.3a (Amp^R^, RSF ori) and pSm.CDF.3a (Sm^R^, CDF ori) were redesigned from the Novagen Duet vectors to generate StarGate® expression constructs compatible with pENTRY (Kan^R^, ColE1 ori) based gene expression in one cell. For the design of the pAmp.RSF basic vector, the following fragments, each containing *Lgu*I recognition sites for specific assembly, were amplified: the RSF ori was derived from pRSFDuet-1 using the primers RSF-ori-s and lacI-as. Blue GCAC was amplified from pENTRY using the primers Blue-GCAC-s and Blue-GCAC-as. The third assembly fragment was termed T7term and was derived from pACYC-Duet-1 using T7term-s and T7term-as as primers and the Amp/Carb resistance gene was derived from pFF.rbs3a (a pNFUSE analog equipped with a high affinity ribosomal binding site rbs3a) using the primers AmpR-s and AmpR-as. The four fragments were assembled in a one pot reaction using *Lgu*I and T4-ligase. The resulting vector pAmp.RSF(blue) was further modified to contain divergent *Esp*3I-in sites for the insertion of gene cassettes from Entry clones as found in StarGate® expression vectors: First, the oligonucleotides LguI(out).3a-s and LguI(out).3a-as were phosphorylated, annealed and subsequently inserted into the *Lgu*I-precut pAmp.RSF(blue) vector, generating pAmp.RSF.3a (white) equipped with *Esp*3I-in sites. To facilitate subcloning steps, lacP/Z was inserted using one of the Blue-T7 cassette PCR amplificates from the aforementioned pFxT7 vector synthesis by means of restriction-ligation with *Esp3*I and T4-ligase, yielding the compatible expression plasmid pAmp.RSF.3a (blue).

The analogous procedure was applied for pSm.CDF.3a, using the following fragments for backbone synthesis: The CDF ori was amplified from pCDFDuet-1 using the primers CDF-ori-s and lacI-as. The Blue GCAC fragment was derived as described for pAmp.RSF.3a. A third fragment, termed SmR-T7term and containing the spectinomycin resistance gene, was amplified from pCDFDuet-1 as well, using the primers T7term-s and SmR-as. The assembly of the three fragments, followed by the equipment with divergent *Esp*3I-in sites and *lacP/Z* insertion, was performed as described for pAmp.RSF.3a. Both compatible plasmids are depicted in Fig. S3c.

### Cloning of *Cupriavidus Necator* Genes

Fragmentation of the 20 genes in question resulted in 33 amplificates using *Cn* genomic DNA (purified using GenElute™ Bacterial Genomic DNA Kit, Sigma) as template. Fragmentation was based on the following considerations: Silent mutations were inserted to remove collectively eight internal *Esp*3I and one *Lgu*I restriction sites (*hoxU*, *hoxY*, *hypA2*, *hypB2*, *hypF2*, *hypE2*, *hypX*, *hoxN1*). In four cases, further silent mutations were inserted to reduce the GC content facilitating primer annealing during PCR (*hoxI*, *hypF2*, *hypC2*, *hypF3*). Genes larger than 1.5 kb were generally split into two or three fragments based on sequencing considerations. For amplification of the 33 fragments, the primers listed in Table S1 were used.

The vector backbone used for acceptance of the fragments during initial *blunt end* insertion was generated via PCR using the primers pFF-(for) and pFF-(rev) with pFF.rbs3a as template. Following purification and phosphorylation using polynucleotide kinase, the fragments were *blunt end* ligated into the linearized pF vector backbone using T4-ligase. The orientation and correct insertion of fragments was determined and two positive clones were sent to sequencing (Eurofins MWG operon) using the primers SQ-360 and SQ-361, respectively. Accurate clones thus obtained were subsequently used to create the Entry clone library, either by fusion of the gene fragments to yield full ORFs or in cases without fragmentation by transfer of the ORF, in each case using *Lgu*I, T4 ligase and pENTRY as the acceptor vector.

### Creation and Fusion of Single Gene Operons Yielding Multi-gene Expression Constructs

Emanating from Entry clones, genes were first subcloned into pFnT7 (upstream) or pFcT7 (downstream) fusion vectors using *Esp*3I and T4 ligase in one pot reactions (analogous procedure as depicted in Fig. S2). The initial fusion of maturation related gene cassettes into pENTRY was performed systematically, assembling *hypA2* and *hypB2*, *hypA3* and *hypB3*, *hypC1* and *hypD1*, *hypC2* and *hypD2*, *hypE1* and *hypF2*, *hypE2* and *hypF3* as well as *hoxW* and *hypX*. For structural genes, *hoxY* and *hoxH* (encoding the H_2_ase module) as well as *hoxF* and *hoxU* (encoding the diaphorase moiety), were assembled separately. *hoxF* and the gene *hoxI* (whose gene product HoxI is HoxFU-associated) were subcloned into both pFxT7(**wt**) and pFxT7(**5′**), aiming at the possibility to create both pseudo-wildtype as well as N-terminally StrepII tagged HoxF or HoxI subunits to facilitate purification of the enzyme variants.

Stepwise assembly of the binary modules was performed analogous to the StarGate® standard fusion process (which, contrary to our system, yields polycistronic operons): Upstream and downstream modules were subcloned into pFF.rbs3a and pFF.c, respectively, and assembled into pENTRY using one pot reactions with either *Lgu*I or *Esp*3I and T4 ligase (analogous procedure as depicted in Fig. S2). For the creation of odd-numbered expression constructs in one vector, upstream fusion partners were fused with pFcT7 (one gene downstream) derivatives as needed.

Compatible acceptor vectors (Fig. S3c) were designed to accept inserts from Entry constructs. Due to the additional feature of blue/white screen capability, these vectors share the basic features with the StarGate® expression vectors (e. g. pPSG, pASG derivatives) with the distinction that they lack regulons for control of polycistonic expression. In order to generate compatible expression constructs, multigene cassettes were transferred from pENTRY to pAmp.RSF.3a or pSm.CDF.3a using one pot restriction-ligation with *Esp*3I and T4 ligase. Sequential transformation of chemically competent cells with multigene constructs (Table S2) yielded the final expression strains (Tables 2 and S3). *E. coli* BL21Star™ (DE3) was used as the basic strain in all expression trials.

### Screening for Structural Genes Bicistronic mRNA Stability and Polycistron Generation

In order to generate polycistronic expression constructs comprising the structural genes *hoxF*, *hoxU*, *hoxY*, *hoxH* and *hoxI*, a screening strategy was applied to determine a functioning order of genes allowing continuous translation. First, the five genes were subcloned into the standard fusion vectors pFF.rbs3a and pFF.c, respectively. Subsequently, five individual polyclonal mixtures were prepared, each containing one distinct pFF.rbs3a derivative and the pFF.c derivatives of all other structural genes. Upon combination with pENTRY in *Lgu*I- and T4 ligase-mediated one step reactions, polyclonal bicistrons were generated. Finally, the five individual plasmid mixtures were subjected to the standard StarGate® subcloning procedure into the expression vector pASG-wt using *Esp*3I and T4 ligase.

The polyclonal pASG-wt derivatives thus obtained were transformed into *E. coli* BL21Star™ (DE3), following plating and incubation over night. On the following day, 25 colonies per plate/mixture were each picked into 600 µL LB medium supplemented with antibiotics (1.5 mL polypropylene tubes) and vigorously shaken at 1300 rpm and 37°C for 4 to 6 hours until cultures reached an OD_578_ of ∼ 0.5. The cells were then pelleted and resuspended in 600 µL of the same medium supplemented with 200 µg·L^−1^ anhydrotetracycline (AHT) followed by a 2 h induction interval in the dark applying the aforementioned growth conditions. Cells were harvested by centrifugation and stored at −20°C until preparation for SDS-PAGE analysis. Following SDS-PAGE [65] and gel staining with Coomassie [66], whole-cell lysates from each sample set were visually analyzed for overproduced proteins in addition to the predetermined first gene product in order. The successful co-production of two subunits indicated a functioning bicistron for later assembly. After determination of a functioning gene order, the SH structural genes were subjected to the standard StarGate® fusion process (Fig. S2), yielding polycistrons with the order *hoxIYHUF*, *hoxYHUF*, *hoxYHU* and *hoxYH*.

### Gene Expression

For multigene co-expression, *E. coli* strain BL21Star™ (DE3) (Invitrogen) was used throughout the study. Expression plasmids were introduced sequentially. The first construct was inserted using heat shock transformation. From the second plasmid on, transformation was performed using TransformAid Bacterial Transformation Kit (Fermentas) according to the manufacturer’s instructions. The main SH production strains generated in this study are listed in Table 2.

Protein expression studies were carried out under ‘autoinduction’ [36] conditions. The basic medium used in these trials was either LB (10 g·L^−1^ tryptone, 5 g·L^−1^ yeast extract, 5 g·L^−1^ NaCl), TB (12 g·L^−1^ tryptone, 24 g·L^−1^ yeast extract, 0.4% (vol/vol) glycerol, 2.3 g·L^−1^ KH_2_PO_4_, 12.5 g·L^−1^ Na_2_HPO_4_), M9 minimal (6 g·L^−1^ Na_2_HPO_4_; 3 g·L^−1^ KH_2_PO_4_; 1 g·L^−1^ NH_4_Cl; 0.5 g·L^−1^ NaCl, 1 mM MgSO_4_, 0.1 mM CaCl_2_, 1 mM thiamine) or a medium modified from a previous expression study [32], termed “hydrogenase expression modified” (HEM) medium (LB basis w/o NaCl, 2 mM MgSO_4_, 50 mL·L^−1^ of 20×phosphate basis (1 M Na_2_HPO_4_, 1 M KH_2_PO_4_ and 0.25 M (NH_4_)_2_SO_4_)). Depending on the conditions and supplementations tested, 0.05–2% (wt/vol) glucose, 1–5% (vol/vol) glycerol and 0.2–0.8% (wt/vol) lactose were added as carbon sources. Where indicated, NiCl_2_ (1–500 µM), FeSO_4_ (25–100 µM), ferric ammonium citrate (25–200 µM), riboflavin (1–10 µM), cysteine (1–5 mM), betaine (2 mM), an amino acid mixture (weighed solid compounds, 1 mM each, dissolved prior use and sterile filtered) or a trace element solution SL6 were supplemented. Antibiotics (kanamycin, spectinomycin, carbenicillin) were added to a final concentration of each 50 mg·L^−1^, except for strains harboring pAmp.RSF.3a-constructs (25 mg·L^−1^ carbenicillin). Media were aseptically prepared, distributed among sterile 500 mL baffled culture flasks (100 mL working volume) and inoculated 1∶50 (vol/vol) from preparatory overnight cultures. Cultivation was carried out at temperatures between 18–37°C with continuous shaking at 100–500 rpm for 20–48 hours. Cells were harvested by centrifugation, washed twice with the buffer used later on for cell opening and stored at −80°C prior use.

For optimized SH production, conditions were as follows: M9 minimal medium was used, supplemented with antibiotics, 0.1% (wt/vol) glucose, 2% (vol/vol) glycerol, 0.8% (wt/vol) lactose, 1 µM NiCl_2_, 100 µM ferric ammonium citrate, 1 µM riboflavin and 5% (vol/vol) LB-medium. 1000 mL baffled culture flasks with a working volume of 550 mL were used. The preparatory culture (100 mL in a 500 mL baffled flask; 300 rpm, 28°C; same medium as main culture, but without lactose supplementation) was inoculated the day before induction from a glycerol stock and was used for inoculation of the induction (main) culture. This transfer was carried out before the preparatory culture reached an OD_578_≥1. Cells were then sedimented by centrifugation (2,000 *g*; 1 min), resuspended (in 50 mL ‘autoinduction’ medium) and added to the induction culture to yield a start-OD_578_ of 0.1. Cultivation was carried out at room temperature (22–25°C) with shaking at 200 rpm for 36–40 hours. In cases where pASG derivatives were combined with the T7 based constructs (SHdec strains; Table 2), autoinduction in was performed likewise and anhydrotetracycline (AHT; 200 µg·L^−1^) was added after 12 to 16 h (OD_578_ of ∼ 0.8–1) to the cultures following combined induction in the dark for further 24 h. Cells were harvested by centrifugation, washed twice with the buffer used later on for cell opening and stored at −80°C prior use.

Anaerobic expression was performed in 2 Liter flasks filled with 1.2 Liter of buffered LB medium supplemented with 100 mM MOPS/KOH pH 7.5, 2 mM cysteine, 2 mM FeSO_4_, 25 µM NiCl_2_, 0.8% (wt/vol) glucose and antibiotics. The flask was sealed with a gas tight butyl rubber stopper fixed with a metal sealing ring and an open top screw cap. Following inoculation to an initial OD_578_ of 0.4, the content was flushed with nitrogen and subsequently gently stirred at 30°C for 2 h. Isopropyl-β-D-thiogalactopyranoside (IPTG) was then added to a final concentration of 1 mM following induction for 20–24 h at 25–30°C and 150 rpm.

### Purification of SH_rec_ and SH_nat_

Native SH was purified from *C. necator* (DSM 428) as described [45] with the following modifications: SH-yield-optimized *Cn* cultivation was carried out in 1 Liter baffled flasks (working volume: 400 mL) with modified FN medium (0.4% fructose; 10×ferric ammonium citrate to a final concentration of 50 mg·L^−1^) for 24 h at 30°C with shaking at 250 rpm. Subsequently, glycerol was added to a final concentration of 0.4% following incubation for further 48 h at room temperature with shaking at 150 rpm. Cells were harvested at an OD_436_ of 12–14 by centrifugation, washed twice with ice-cold 50 mM KPi buffer (pH 7.0) containing 50 mM succinate and stored at −80°C prior use.

20 grams of packed cells thus obtained were used for purification of wildtype SH with the protocol recently described [45], except that only 10 mM ferricyanide were added for oxidation of the 35% ammonium sulfate supernatant and a DEAE-Sepharose column was used for ion exchange chromatography. Buffers were supplemented with 2 µM FMN and all purification steps were carried out at room temperature.

Recombinant SH variants were purified by StrepTactin affinity chromatography using strains SH1F (four-subunit SH with 5′-tagged HoxF) and SH2F (six-subunit SH with 5′-tagged HoxI) for enzyme production. In contrast to purification of the native enzyme, the recombinant SH preparations listed in Tables 3 and 4 were purified focusing on purity rather than yield. Approximately 6–8 grams of cells obtained from a 500 mL ‘autoinduction culture’ (see above) were resuspended in 2 mL 50 mM KPi buffer (pH 7.0) per gram cells containing 5 mM MgCl_2_, 50 mM succinate, DNase, 2 µM FMN, 25 µg avidin per gram cells and 0.05 mM PMSF and disrupted by four consecutive freeze/thaw cycles. Unbroken cells, debris and membranes were removed by ultracentrifugation at 140,000 *g* and 2°C for 45 min. The soluble extract was loaded onto a pre-equilibrated 5 mL Strep-Tactin Superflow gravity flow column, following 5 column volumes (CV) washing with 50 mM KPi buffer (pH 7.0) and elution with the same buffer containing 2.5 mM Desthiobiotin. SH-containing fractions were pooled, concentrated and loaded onto a 50 mM KPi buffer (pH 7.0) pre-equilibrated Superdex 200 HR 10/300 gel filtration column for polishing.

The homogeneity of preparations was routinely analyzed by SDS-PAGE [65], [66]. Protein concentrations were determined by the method of Bradford [67].

Colorimetric determination of non-heme iron in purified SH variants was carried out according to [68]. For UV/Vis spectroscopy, an Ultrospec 3000 spectrophotometer (Pharmacia) was used. Sodium dithionite (Sigma; 85%) was recrystallized twice under anoxic conditions by adapting a standardized protocol [69]. Final purity of the crystallized dithionite was 96% as determined by correlation of the mass to the absorbance at 315 nm (ε_315_ = 8.0 mM^−1^·cm^−1^).

### Hydrogenase Activity Measurements

Hydrogenase H_2_:NAD^+^physiological activity was measured in an anaerobic glove box (2.5–5% H_2_; rest N_2_) following the NADH dependent increase in absorbance spectrophotometrically at 340 nm in a 1 cm cuvette. The assay mixture (1 mL total volume) contained 20 µL of enzyme sample, 1 µM FMN, 5 µM NADH and 50 mM Tris buffer pH 8.0 equilibrated with the glove box atmosphere (dissolved 19–38 µM H_2_). The reaction was started by addition of NAD^+^to a final concentration of 5 mM.

For aerobic measurements, 800–900 µL of an air-saturated 50 mM Tris buffer pH 8.0 was used instead of the buffer saturated with the glove-box atmosphere. All compounds except for NAD^+^and H_2_ were combined. Then, 50 mM Tris buffer pH 8.0 saturated with 80% H_2_ (rest N_2_) was added to the desired final H_2_-concentration and the volume filled up to 1 mL. The cuvette was sealed with a gas-tight stopper and the reaction was subsequently started by addition of NAD^+^to a final concentration of 5 mM.

An extinction coefficient of ε _340 nm_ = 6.22 mM^−1^·cm^−1^ was used for NADH. 1 Unit is defined as the H_2_-mediated reduction of 1 µmol NAD^+^per minute.

## Supporting Information

Figure S1 **General scheme for the basic cloning procedure used throughout this study.** The classical procedure for expressing one gene of interest (GOI) in a designated expression vector pX is depicted. The system operates alternating between *Lgu*I- and *Esp*3I-mediated restriction-ligation, performed in one step reactions. Initially, the GOI is amplified with primers attaching *Lgu*I-sites to the amplificate, which allows subsequent transfer into pENTRY. From this point, subcloning of the GOI into fusion or expression vectors is readily accomplished. The overhangs created via restriction digest are shown in red and cutting positions are indicated with black triangles. The expression construct leaves no further restriction sites. Acceptor vectors contain *lacP/Z* inserts which are replaced by the GOI in the course of the transfer (blue/white screen). Note that the *Cn* fragments cloned in this study were initially *blunt end* inserted into a linearized pF backbone prior transfer/fusion of the full ORF into pENTRY, which is not shown here (see methods section of the paper).
(TIF)

Figure S2 **Fusion cloning of two genes of interest.** The system operates alternating between *Lgu*I- and *Esp*3I-mediated subcloning, performed in one step reactions. Gene 1 is subcloned into an upstream fusion vector. This may be pFF.rbs3a (pNFUSE in StarGate® system) for classical assembly of polycistrons as shown in this scheme, or the newly designed pFnT7 plasmids (see Fig. S3a,b). Gene 2 is subcloned into a respective downstream fusion vector, which can either be pFF.c (pCFUSE in StarGate® system) or pFcT7 in the new system. Upstream and downstream constructs are used for simultaneous fusion of the inserts into pENTRY in a one step reaction. As stated before, the fusion scheme is analogous for creation of T7-promoter and –terminator flanked multiple gene cassettes with pFxT7 derivatives, except that the ribosomal binding site is superfluous. After fusion of genes or cassettes into pENTRY, this step can be repeated as needed to yield the multigene constructs. The overhangs created via restriction digest are shown in red and cutting positions are indicated with black triangles.
(TIF)

Figure S3 **New vectors synthesized in this study.** **a**) pFnT7-series, which represent the upstream fusion vectors for assembly of multiple gene cassettes; **b**) pFcT7-series, which represent the downstream fusion vectors for assembly of multiple gene cassettes; Both sets of vectors are designed for acceptance of genes from pEntry constructs. Placed in the pFxT7-vectors, the individual genes are automatically equipped with a T7-promoter and –terminator. **c**) Combinatorial vectors, allowing co-expression of genes from different constructs in one cell; pEntry (ColE1 origin; kanamycin resistance gene, Kan^R^) is a standard fusion vector and a high copy number plasmid (40 cell^−1^), which was already available at the beginning of the study. pSm.CDF.3a (CDF origin; spectinomycin resistance gene, Sm^R^) is a vector with moderate copy number (20–40 cell^−1^). In this study, this vector served in most strains as the carrier of the maturation modules M1/M2. pAmp.RSF.3a (RSF origin; ampicillin/carbenicillin resistance gene, Amp^R^) contains the RSF origin and, therefore, exhibits the highest copy number (>100 cell^−1^). It was used in this study as a carrier of modules in combination with the other two compatible plasmids. Relevant *Lgu*I and *Esp*3I restriction sites for the cloning step are indicated. The pFxT7 vectors (a, b) accept inserts from pEntry constructs by *Esp*3I/T4-ligase mediated transfer and allow subsequent fusion of thus generated gene cassettes with the respective fusion construct by *Lgu*I/T4-ligase mediated transfer into pEntry. pSm.CDF.3a and pAmp.RSF.3a (c) were designed as acceptor vectors of genes/modules from pEntry constructs by *Esp*3I/T4-ligase mediated transfer.
(TIF)

Figure S4 **Effect of extracellular nickel concentrations on maturation efficiency.** NiCl_2_ was supplemented as indicated. Strains: K1A (pSH4.wt and pM1); K1A deltaHypA2B2 (pSH4.wt and pM1Δ*hypA2B2*); K1A–HoxN1 (pSH4.wt and pM1-*hoxN1*); K1B (pSH4.wt and pM2); K1B–HoxN1 (pSH4.wt and pM2-*hoxN1*). The effect given by the omission of the HypA2B2 complex could not be fully complemented by elevating nickel concentrations in the medium. Strains containing an additional gene *hoxN1*, which encodes a high-affinity nickel permease, showed highly increased maturation efficiency. However, the stimulating effect was observable at low nickel concentrations (1 µM), while an adverse effect was observable at higher concentrations, where cells are probably intoxicated by elevated intracellular nickel levels.
(TIF)

Figure S5 **Molecular mass determination of the two recombinant SH variants by analytical size exclusion chromatography (gel filtration).** Molecular masses (MW) were plotted on a logarithmic scale. Standards and samples were analyzed on a Superdex 200 HR 10/300 gel filtration column, pre-equilibrated with 50 mM KPi buffer pH 7.0. The following proteins were used as standards: thyroglobulin (669 kDa), ferritin (440 kDa), catalase (232 kDa), aldolase (158 kDa), bovine serum albumin (67 kDa). The formula for the linear fit was K_AV_  =  –0,137·ln(MW) + 1,2185 (R^2^  =  0.982). The calculated masses of the two variants were 171 kDa (**1**, four-subunit SHvar1) and 213 kDa (**2**, six-subunit SHvar2).
(TIF)

Table S1 **List of all primers and oligonucleotides used throughout this study.**
(DOCX)

Table S2 **List of important strains and plasmids used or generated in this study.**
(DOCX)

Table S3 **Recombinant** ***E. coli*** **strains and activities for maturation studies (deletion and substitution).**
(DOCX)

Table S4 **Selection of three independent purification trials for determination of the ideal buffer composition for SH stabilization in** ***E. coli*** **extracts.** In each case, 1 gram of wet-packed cells (strain SH1F; four-subunit SH variant 1) from the same ‘autoinduction’ batch was used for purification.
(DOCX)

Table S5 **Properties of the native and recombinant SH variants purified in this study.**
(DOCX)

Table S6 **Activation behavior and kinetic properties of native and recombinant SH preparations upon application of different assay conditions.**
(DOCX)
