# Supplementary figures and images for: An Innovative Cloning Platform Enables Large-Scale Production and Maturation of an Oxygen-Tolerant [NiFe]-Hydrogenase from Cupriavidus necator in Escherichia coli
Source: PLoS One. 2013 Jul 5;8(7):e68812. doi: 10.1371/journal.pone.0068812 (PMC3702609; doi:10.1371/journal.pone.0068812)

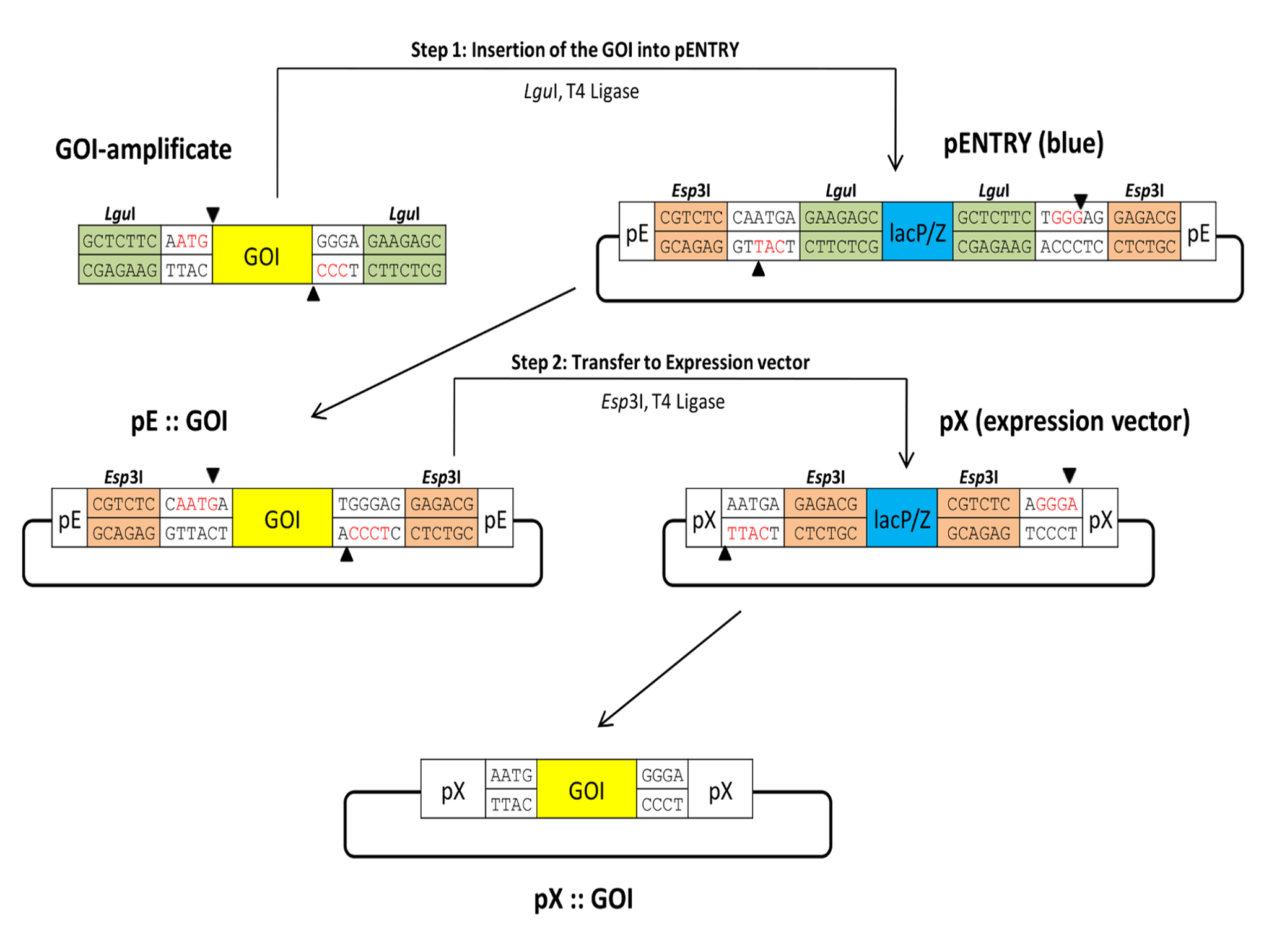

Supplement: Figure S1 — General scheme for the basic cloning procedure used throughout this study. The classical procedure for expressing one gene of interest (GOI) in a designated expression vector pX is depicted. The system operates alternating between LguI- and Esp3I-mediated restriction-ligation, performed in one step reactions. Initially, the GOI is amplified with primers attaching LguI-sites to the amplificate, which allows subsequent transfer into pENTRY. From this point, subcloning of the GOI into fusion or expression vectors is readily accomplished. The overhangs created via restriction digest are shown in red and cutting positions are indicated with black triangles. The expression construct leaves no further restriction sites. Acceptor vectors contain lacP/Z inserts which are replaced by the GOI in the course of the transfer (blue/white screen). Note that the Cn fragments cloned in this study were initially blunt end inserted into a linearized pF backbone prior transfer/fusion of the full ORF into pENTRY, which is not shown here (see methods section of the paper). (TIF) [file pone.0068812.s001.tif]

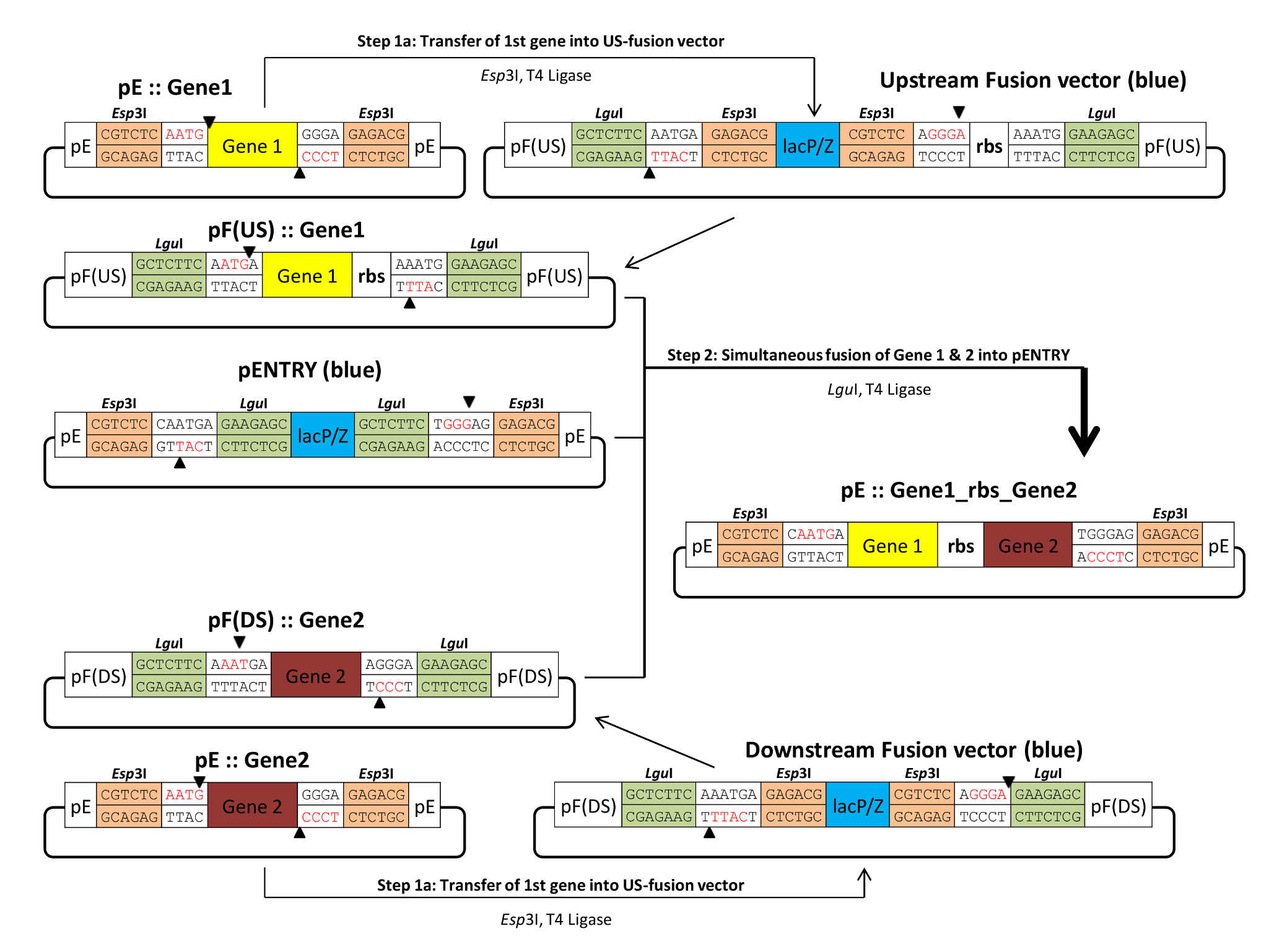

Supplement: Figure S2 — Fusion cloning of two genes of interest. The system operates alternating between LguI- and Esp3I-mediated subcloning, performed in one step reactions. Gene 1 is subcloned into an upstream fusion vector. This may be pFF.rbs3a (pNFUSE in StarGate® system) for classical assembly of polycistrons as shown in this scheme, or the newly designed pFnT7 plasmids (see Fig. S3a,b). Gene 2 is subcloned into a respective downstream fusion vector, which can either be pFF.c (pCFUSE in StarGate® system) or pFcT7 in the new system. Upstream and downstream constructs are used for simultaneous fusion of the inserts into pENTRY in a one step reaction. As stated before, the fusion scheme is analogous for creation of T7-promoter and –terminator flanked multiple gene cassettes with pFxT7 derivatives, except that the ribosomal binding site is superfluous. After fusion of genes or cassettes into pENTRY, this step can be repeated as needed to yield the multigene constructs. The overhangs created via restriction digest are shown in red and cutting positions are indicated with black triangles. (TIF) [file pone.0068812.s002.tif]

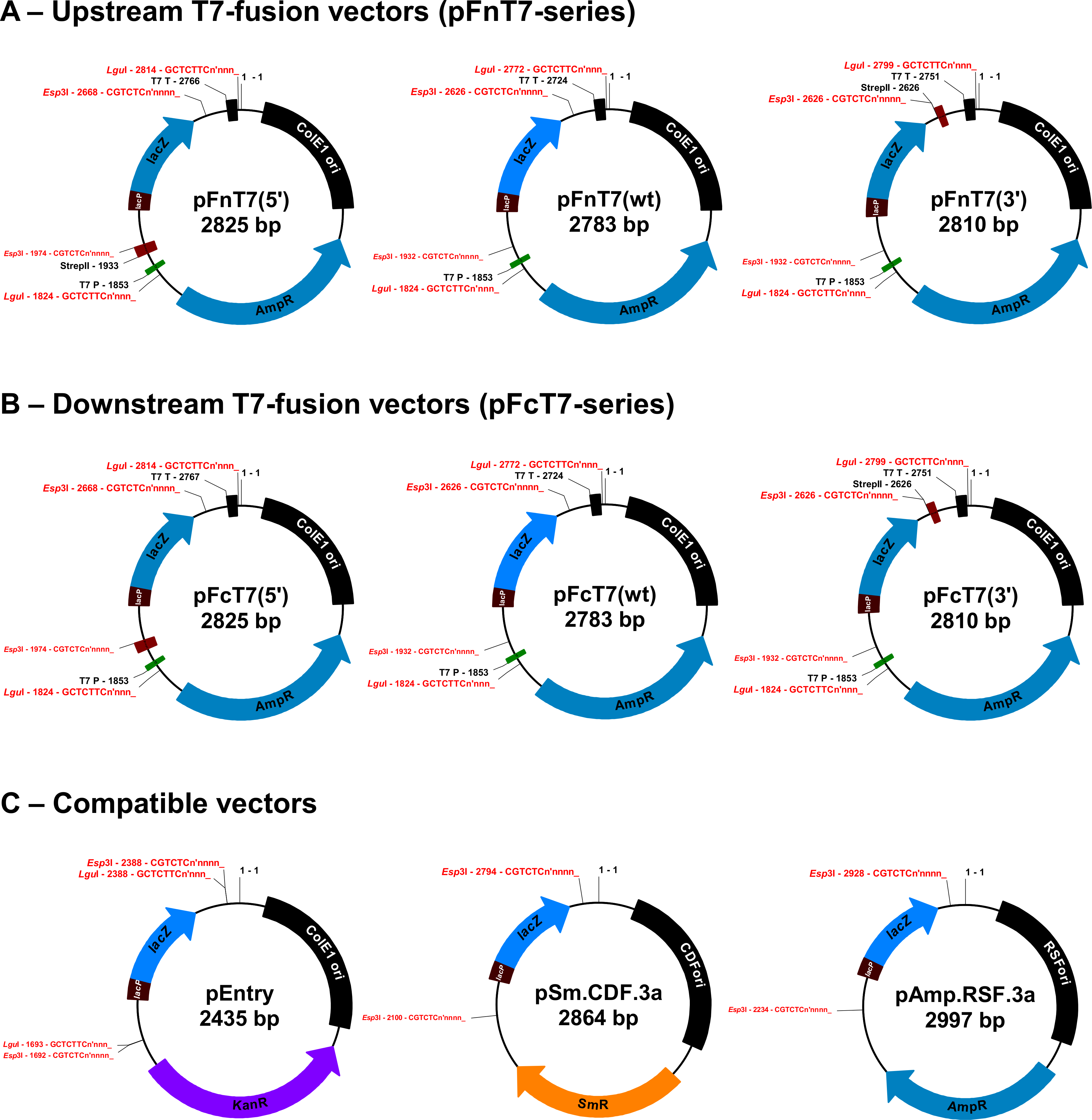

Supplement: Figure S3 — New vectors synthesized in this study. a) pFnT7-series, which represent the upstream fusion vectors for assembly of multiple gene cassettes; b) pFcT7-series, which represent the downstream fusion vectors for assembly of multiple gene cassettes; Both sets of vectors are designed for acceptance of genes from pEntry constructs. Placed in the pFxT7-vectors, the individual genes are automatically equipped with a T7-promoter and –terminator. c) Combinatorial vectors, allowing co-expression of genes from different constructs in one cell; pEntry (ColE1 origin; kanamycin resistance gene, KanR) is a standard fusion vector and a high copy number plasmid (40 cell−1), which was already available at the beginning of the study. pSm.CDF.3a (CDF origin; spectinomycin resistance gene, SmR) is a vector with moderate copy number (20–40 cell−1). In this study, this vector served in most strains as the carrier of the maturation modules M1/M2. pAmp.RSF.3a (RSF origin; ampicillin/carbenicillin resistance gene, AmpR) contains the RSF origin and, therefore, exhibits the highest copy number (>100 cell−1). It was used in this study as a carrier of modules in combination with the other two compatible plasmids. Relevant LguI and Esp3I restriction sites for the cloning step are indicated. The pFxT7 vectors (a, b) accept inserts from pEntry constructs by Esp3I/T4-ligase mediated transfer and allow subsequent fusion of thus generated gene cassettes with the respective fusion construct by LguI/T4-ligase mediated transfer into pEntry. pSm.CDF.3a and pAmp.RSF.3a (c) were designed as acceptor vectors of genes/modules from pEntry constructs by Esp3I/T4-ligase mediated transfer. (TIF) [file pone.0068812.s003.tif]

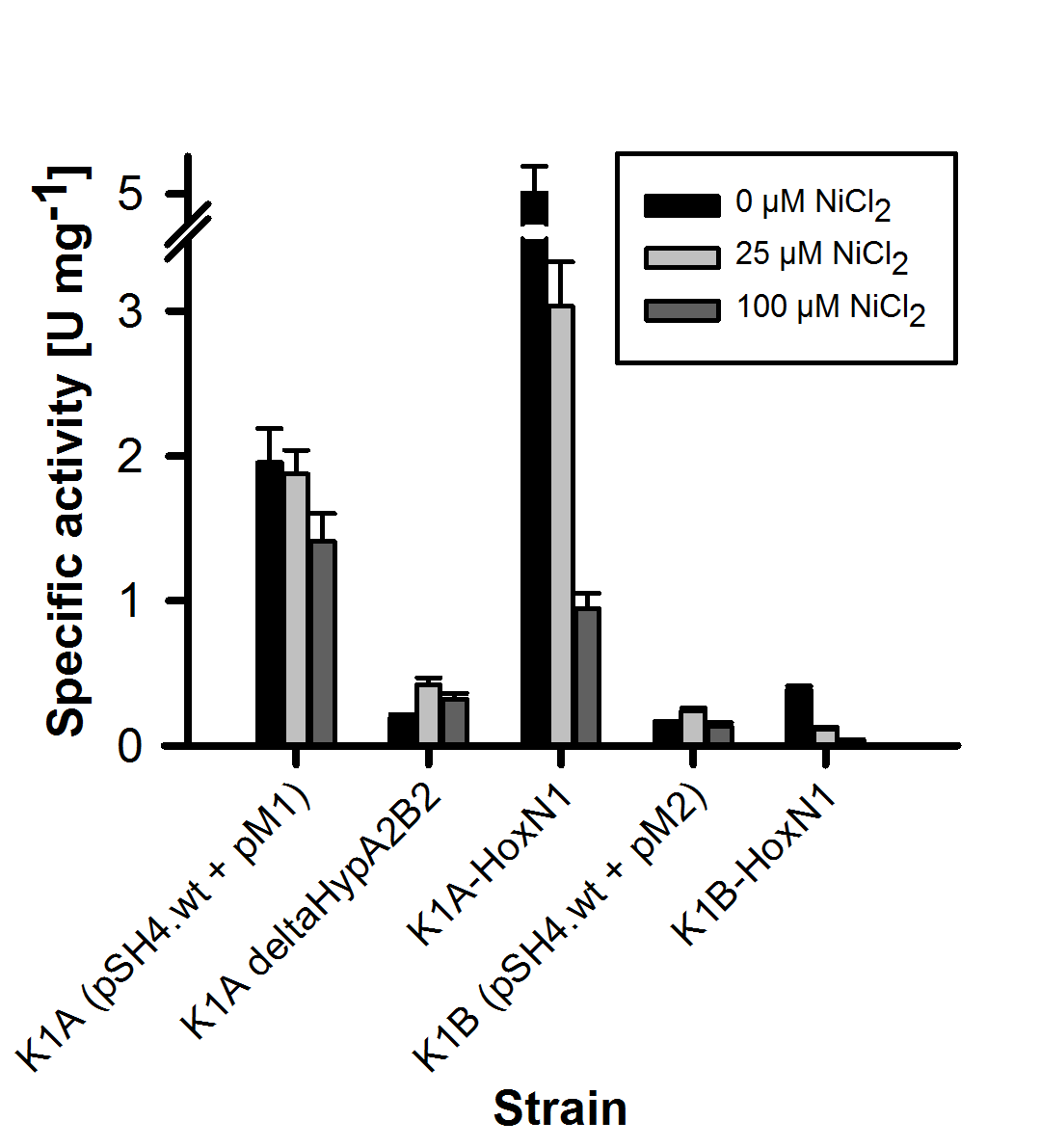

Supplement: Figure S4 — Effect of extracellular nickel concentrations on maturation efficiency. NiCl2 was supplemented as indicated. Strains: K1A (pSH4.wt and pM1); K1A deltaHypA2B2 (pSH4.wt and pM1ΔhypA2B2); K1A–HoxN1 (pSH4.wt and pM1-hoxN1); K1B (pSH4.wt and pM2); K1B–HoxN1 (pSH4.wt and pM2-hoxN1). The effect given by the omission of the HypA2B2 complex could not be fully complemented by elevating nickel concentrations in the medium. Strains containing an additional gene hoxN1, which encodes a high-affinity nickel permease, showed highly increased maturation efficiency. However, the stimulating effect was observable at low nickel concentrations (1 µM), while an adverse effect was observable at higher concentrations, where cells are probably intoxicated by elevated intracellular nickel levels. (TIF) [file pone.0068812.s004.tif]

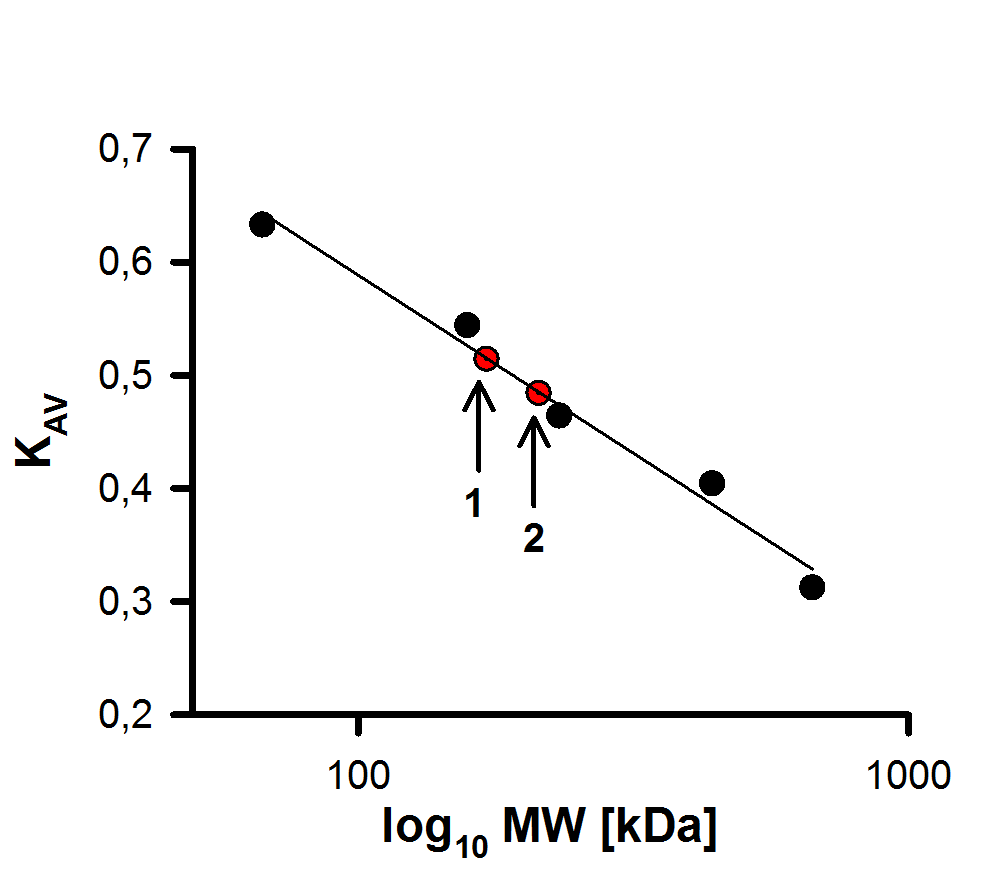

Supplement: Figure S5 — Molecular mass determination of the two recombinant SH variants by analytical size exclusion chromatography (gel filtration). Molecular masses (MW) were plotted on a logarithmic scale. Standards and samples were analyzed on a Superdex 200 HR 10/300 gel filtration column, pre-equilibrated with 50 mM KPi buffer pH 7.0. The following proteins were used as standards: thyroglobulin (669 kDa), ferritin (440 kDa), catalase (232 kDa), aldolase (158 kDa), bovine serum albumin (67 kDa). The formula for the linear fit was KAV = –0,137·ln(MW) + 1,2185 (R2 = 0.982). The calculated masses of the two variants were 171 kDa (1, four-subunit SHvar1) and 213 kDa (2, six-subunit SHvar2). (TIF) [file pone.0068812.s005.tif]
