# Supplementary material for: An Innovative Cloning Platform Enables Large-Scale Production and Maturation of an Oxygen-Tolerant [NiFe]-Hydrogenase from Cupriavidus necator in Escherichia coli
Source: PLoS One. 2013 Jul 5;8(7):e68812. doi: 10.1371/journal.pone.0068812 (PMC3702609; doi:10.1371/journal.pone.0068812)
Supplement: Table S1 — List of all primers and oligonucleotides used throughout this study. (DOCX) [file pone.0068812.s006.docx]

**Table S1.** List of all primers and oligonucleotides used throughout this study.

| **Category** | **Locus tag / Vector template** | **Fragment** | **Gene / Fragment** | **Primer name** | **Primer sequence** |
| --- | --- | --- | --- | --- | --- |
| **Primers for linear pF-backbone used for blunt-end insertion of *Cn* gene fragments** | pFF.rbs3a |  | pF backbone | pFF-(for) | GGAAGAGCTCCAAAGGCGG |
|  |  |  |  | pFF-(rev) | TGAAGAGCTGGCACTTTTCGG |
| **Sequencing primers** |  |  |  | SQ-360 | GGGTTATTGTCTCATGAGCG |
|  |  |  |  | SQ-361 | GAGCGTCGATTTTTGTGATGC |
| **Primers for *Cn* gene amplification** | PHG0**88** | **88.1** | *hoxF* | PHG088.1 s | ATGGATAGTCGTATCACGACAATAC |
|  |  |  |  | PHG088.1 as | AAGCTGTCGCTCCAGATAATCCTTGAG |
|  |  | **88.2** |  | PHG088.2 s | CTTCAGGAACTTCGGGAGGACGGG |
|  |  |  |  | PHG088.2 as | CCCTCATCTTGTCACCTCTTCCAGATCCTTCAG |
|  | PHG0**89** | **89.1** | *hoxU* | PHG089.1 s | ATGAGCATTCAAATTACGATCGACGGCAAG |
|  |  |  |  | PHG089N.1 as | TTTTCTCGCAACTCGGGCAGTTGTG |
|  |  | **89.2** |  | PHG089N.2 s | AAAGCGGCCGTTGCCAGTTGCAG |
|  |  |  |  | PHG089.2 as | CCCTCATTTGTCTTCTCCTTCCAGCGCGCG |
|  | PHG0**90** | **90.1** | *hoxY* | PHG090.1 s | ATGAGAGCCCCCCACAAAGACG |
|  |  |  |  | PHG090.1 as | CTCCACCTTCCACGAAGCCGATC |
|  |  | **90.2** |  | PHG090.2 s | GAGTCTCGAGCGAGGAGAACATCG |
|  |  |  |  | PHG090.2 as | CCCTCAATCGTAGCGATTGATCGAGCTCG |
|  | PHG0**91** |  | *hoxH* | PHG091 s | ATGAGCAGAAAACTGGTTATCGACCCGGTG |
|  |  |  |  | PHG091 as | CCCTCAGCGGGCGCGTTCATCGATC |
|  | PHG0**93** |  | *hoxI* | PHG093 s | ATGAAAGAGCAGGAAATCGACAGG |
|  |  |  |  | PHG093 asN | CCCTAACCTCGTCCTCTACCACCCGTCGAGATGTACTCTTGC |
|  | PHG0**15** |  | *hypC1* | PHG015 s | ATGTGCCTAGCGATTCCCGC |
|  |  |  |  | PHG015 as | CCCTCATGCCGGCTCCTGATGAATG |
|  | PHG0**16** |  | *hypD1* | PHG016 s | ATGAAATACATCGAAGAATTTCGCGACGGC |
|  |  |  |  | PHG016 as | CCCTCATGCTGCCACCAAGGGGATATC |
|  | PHG0**17** |  | *hypE1* | PHG017 s | ATGAGCGGCACCGTTAAACTGGGC |
|  |  |  |  | PHG017 as | CCCTCAACAAATGCGCGGAAGCTGCTCG |
|  | PHG0**18** | **18.1** | *hypX* | PHG018.1 s | ATGCGCATATTGCTCCTCACCCATAGC |
|  |  |  |  | PHG018.1 as | ATCTCGAACGATCGCCCAGTCCAG |
|  |  | **18.2** |  | PHG018.2 s | GATGAACGAAGTTGGGGTGTGACGGTG |
|  |  |  |  | PHG018.2 as | AAATCCTCTCCCCCCAGCAGCAC |
|  |  | **18.3** |  | PHG018.3 s | TTTTTTCAGCAATGGTATTCATCTGCACCAAATCG |
|  |  |  |  | PHG018.3 as | CCCTCAAGATCGTTTCCCCGCAAGTGC |
|  | PHG0**23** | **23.1** | *hoxN1* | PHG023.1 s | ATGTTCCAATTGCTCGCTGGCGTACG |
|  |  |  |  | PHG023.1 as | TTCAATGCCACCGACGATGAGGGC |
|  |  | **23.2** |  | PHG023.2 s | GAAACGCTTGGCCTCCTGGCC |
|  |  |  |  | PHG023.2 as | CCCTCAGGCACGCACTTCGCTGTC |
|  | PHG0**72** | **72.1** | *hypF3* | PHG072.1 s | ATGCGAGGAATGGTACAAGGAGTAGGATTCCGGCCCACCGTATGGCG |
|  |  |  |  | PHG072.1 as | CAACCCACATTCGGCCGCGGTC |
|  |  | **72.2** |  | PHG072.2 s | TTGCTGCAAAGCCCGGCGGC |
|  |  |  |  | PHG072.2 as | AGCAGGCCAGCCGCGTGAAG |
|  |  | **72.3** |  | PHG072.3 s | GCTTCAAGCCCGTGGGGATGC |
|  |  |  |  | PHG072.3 as | CCCCTAGGCACATGTTGTACTCCGATAGCCC |
|  | PHG0**73** |  | *hypC2* | PHG073 s | ATGTGCCTAGGTATCCCCGGACAGATCC |
|  |  |  |  | PHG073 as | CCCCTATGGTGGTGCTGGTGACCCCGCGCGCATCTCGTC |
|  | PHG0**75** |  | *hypD2* | PHG075 s | ATGAAGTACGTCGACGAATTCCGCGATCCC |
|  |  |  |  | PHG075 as | CCCTCATTCATGGCCGGCTCCGGC |
|  | PHG0**76** | **76.1** | *hypE2* | PHG076.1 s | ATGAATGACCGTGTTCCGGTCCCG |
|  |  |  |  | PHG076.1 as | ACATCTCCCGCACGCGCATG |
|  |  | **76.2** |  | PHG076.2 s | TGTGGTTCTCGTCAATGGCTATCTTGGCGAC |
|  |  |  |  | PHG076.2 as | CCCTCAGCAGATCCGCGGCAGCTG |
|  | PHG0**77** |  | *hypA3* | PHG077 s | ATGCACGAACTAAGTATCGCGAACAGCGTGG |
|  |  |  |  | PHG077 as | CCCTCACGCCACCTCCATGCGCC |
|  | PHG0**78** |  | *hypB3* | PHG078 s | ATGTGCACGACTTGCGGTTGCG |
|  |  |  |  | PHG078 as | CCCTCACGCCGGAAACGAGTCCTCAG |
|  | PHG0**92** |  | *hoxW* | PHG092 s | ATGAACGCGCCCGCTGAGTTTC |
|  |  |  |  | PHG092 as | CCCTCACGAGGTTTGACGCTCGGC |
|  | PHG0**94** | **94.1** | *hypA2* | PHG094.1 s | ATGCATGAGATGTCGCTGGCCG |
|  |  |  |  | PHG094.1 as | TTCGCTGCAGTGCAGGCACC |
|  |  | **94.2** |  | PHG094.2 s | GAAACGGTGGCGATCGGAGCG |
|  |  |  |  | PHG094.2 as | CCCTCACGCCACTTCCAGATCCATGACG |
|  | PHG0**95** | **95.1** | *hypB2* | PHG095.1 s | ATGTGCACCAACTGTGGCTGC |
|  |  |  |  | PHG095.1 as | TACGAAGTTCAGCGCGAACACGC |
|  |  | **95.2** |  | PHG095.2 s | GTATCGAGCCCCGGCTCCGG |
|  |  |  |  | PHG095.2 as | CCCCTACACCTTGAGCGCCGCCAGTTG |
|  | PHG0**96** | **96.1** | *hypF2* | PHG096.1 s | ATGCTGATGCCGCGCCGTCC |
|  |  |  |  | PHG096.1 as | TTCGGCGATCGGATCAACGTCCTCGAC |
|  |  | **96.2** |  | PHG096.2 s | GAAACGGTGGCGCGCCTGCAG |
|  |  |  |  | PHG096.2 as | TACGAAGGCCTCGTCGCCGC |
|  |  | **96.3** |  | PHG096.3 s | GTATCTCAGCATGTGGGCGATCTCGGC |
|  |  |  |  | PHG096.3 as | CCCTCAATTTGGTGCTCGTTGCAGCGCCACCCAGACCTG |
| **Primers for construction of compatible plasmids** | pACYCDuet-1 |  | p15Aori | p15Aori-s | AACGTCTCACGAGCATTTTCGCCAAAAGTTGGCC |
|  |  |  |  | lacI-as | AACGTCTCAGCAGTCTGTCAGTGAAGCTTGCGCAACGCAATTAATGTAAGTTAG |
|  |  |  | Cam^R^-1 | T7term-s | AACGTCTCAGCACTCTGTCACTGAAGCTTCTGAGCAATAACTAGCATAACCCCTTGG |
|  |  |  |  | CamR-as1 | AACGTCTCATCGCAGCCAATCCCTGGGTGAGTTTC |
|  |  |  | Cam^R^-2 | CamR-s2 | AACGTCTCAGCGACGAAAAACATATTCTCAATAAACCCTTTAGG |
|  |  |  |  | CamR-as2 | AACGTCTCACTCGAGGACGTTGATCGGCACGTAAG |
|  |  |  | T7term | T7term-s | AACGTCTCAGCACTCTGTCACTGAAGCTTCTGAGCAATAACTAGCATAACCCCTTGG |
|  |  |  |  | T7term-as | AACGTCTCAGAATTAATTCATGAGCGGATACATATTTGAATG |
|  | pCDFDuet-1 |  | CDF ori | CDFori-s | AACGTCTCCCGAGAGCTACGCTCCGGGCGTGAGACTGC |
|  |  |  |  | lacI-as | AACGTCTCAGCAGTCTGTCAGTGAAGCTTGCGCAACGCAATTAATGTAAGTTAG |
|  |  |  | Sm^R^-T7term | T7term-s | AACGTCTCAGCACTCTGTCACTGAAGCTTCTGAGCAATAACTAGCATAACCCCTTGG |
|  |  |  |  | SmR-as | AACGTCTCCCTCGAGGGAGCGTAGCGACCGAGTGAG |
|  | pENTRY (blue) |  | Kan^R^ | KanR-s | AACGTCTCCCTCGAGGTGGCACTTTTCGGGGAAATGTG |
|  |  |  |  | KanR-as | AACGTCTCCATTCTCAGAAGAACTCGTCAAGAAGGCG |
|  |  |  | Blue-GCAC | Blue-GCAC-s | AACGTCTCCGTGCAAGAAGAGCCTGCAGCCCAATACGC |
|  |  |  |  | Blue-GCAC-as | AACGTCTCCCTGCCAGAAGAGCCTCGAGGTGAACC |
|  | pFF.c (blue) |  | ColE1 ori | ColE1-s | AACGTCTCACGAGAGGGTGAAGATCCTTTTTGATAATCTCATGAC |
|  |  |  |  | ColE1-as | AACGTCTCAGCAGTCTGTCAGTGAAGCTTGCGGTAATACGGTTATCCACAG |
|  |  |  | Amp^R^ | AmpR-s | AACGTCTCCCTCGAGCACTTTTCGGGGAAATGTGCG |
|  |  |  |  | AmpR-as | AACGTCTCCATTCTACCAATGCTTAATCAGTGAGGC |
|  | pRSFDuet-1 |  | RSF ori | RSFori-s | AACGTCTCCCGAGAGCATGCAGCACTCTTCCGCTTCCTCGCTCACTG |
|  |  |  |  | lacI-as | AACGTCTCAGCAGTCTGTCAGTGAAGCTTGCGCAACGCAATTAATGTAAGTTAG |
| **pFxT7 fusion vector primers** | pPSG(wt) /  pPSG(3'-StrepII) / pPSG(5'-StrepII) |  | T7 cassette 3'/5'/wt pFn1 | T7cas_pFn1 s | AAGCTCTTCAATGAATGCAGGATCTCGATCCCGC |
|  |  |  |  | T7cas_pFn1 as | AAGCTCTTCCATTCTTTCAGCAAAAAACCCCTCAAGACCCG |
|  |  |  | T7 cassette 3'/5'/wt pFc1 | T7cas_pFc1 s | AAGCTCTTCAAATAATGCAGGATCTCGATCCCGC |
|  |  |  |  | T7cas_pFc1 as | AAGCTCTTCTCCCCTTTCAGCAAAAAACCCCTCAAGACCCG |
| **Oligonucleotides** |  |  | LguI(out).2 | LguI(out).2 s | GCAGCTCTTCAGCAATGAGAGACGTACGTCTCAGGGAGGCAGAAGAGC |
|  |  |  |  | LguI(out).2 as | GCCGCTCTTCTGCCTCCCTGAGACGTACGTCTCTCATTGCTGAAGAGC |
|  |  |  | LguI(out).3a | LguI(out).3a s | GCCGCTCTTCAGCAATGAGAGACGTACGTCTCAGGGAGGCAGAAGAGC |
|  |  |  |  | LguI(out).3a as | GCAGCTCTTCTGCCTCCCTGAGACGTACGTCTCTCATTGCTGAAGAGC |

**Sequence color code:** Green = *Esp*3I recognition site; Red = *Lgu*I recognition site; Blue = Predesigned *Lgu*I-mediated overhangs in gene fragments for specific insertion into pEntry and (where necessary) for simultaneous fusion of gene fragments to the full ORF.
