# Supplementary material for: An Innovative Cloning Platform Enables Large-Scale Production and Maturation of an Oxygen-Tolerant [NiFe]-Hydrogenase from Cupriavidus necator in Escherichia coli
Source: PLoS One. 2013 Jul 5;8(7):e68812. doi: 10.1371/journal.pone.0068812 (PMC3702609; doi:10.1371/journal.pone.0068812)
Supplement: Table S2 — List of important strains and plasmids used or generated in this study. (DOCX) [file pone.0068812.s007.docx]

**Table S2.** List of important strains and plasmids used or generated in this study.

| **Strains & plasmids** | | |
| --- | --- | --- |
| **Strains** | **Genotype / relevant characteristics** | **Source** |
| *Cupriavidus necator* (form. *Ralstonia eutropha* H16) | wildtype; SH^+^, MBH^+^ | DSM428,  ATCC 17699 |
| *E. coli* DH5α | *fhuA2* Δ*(argF-lacZ)U169 phoA glnV44 Φ80* Δ*(lacZ)M15 gyrA96 recA1 relA1 endA1 thi-1 hsdR17* | Invitrogen |
| *E. coli* BL21Star™ (DE3) | *dcm ompT hsdS*(r_B_^-^m_B_^-^) *gal* | Invitrogen |
| *E. coli* BL21Star™ (DE3) (expression strains; see Tables 2 & S3) | *dcm ompT hsdS*(r_B_^-^m_B_^-^) *gal*; contain plasmids as listed *vide infra* | This study |
| **Plasmids** | | |
| **a. Original vectors** | | |
| pE / pENTRY | cloning & compatible expression vector; Kan^R^; ColE1 ori; lacP/Z | T. Selmer |
| pFF.rbs3a | std. & polycistronic US fusion vector; Amp^R^; lacP/Z | T. Selmer |
| pFF.c | std. & polycistronic DS fusion vector; Amp^R^; lacP/Z | T. Selmer |
| pFn1 | pFF.rbs3a backbone; *Lgu*I insertion site; US-orientation; Amp^R^; lacP/Z | T. Selmer |
| pFc1 | pFF.rbs3a backbone; *Lgu*I insertion site; DS-orientation; Amp^R^; lacP/Z | T. Selmer |
| pASG(wt) | std. expression vector; pseudo-wt; Amp^R^; lacP/Z | T. Selmer |
| pASG(3') | std. expression vector; 3'-StrepII-tag; Amp^R^; lacP/Z | T. Selmer |
| pASG(5') | std. expression vector; 5'-StrepII-tag; Amp^R^; lacP/Z | T. Selmer |
| pPSG(wt) | std. expression vector; pseudo-wt; Amp^R^; lacP/Z | T. Selmer |
| pPSG(3') | std. expression vector; 3'-StrepII-tag; Amp^R^; lacP/Z | T. Selmer |
| pPSG(5') | std. expression vector; 5'-StrepII-tag; Amp^R^; lacP/Z | T. Selmer |
| pACYCDuet-1 | Duet expression vector; Cam^R^ | Novagen |
| pCDFDuet-1 | Duet expression vector; Sm^R^ | Novagen |
| pRSFDuet-1 | Duet expression vector; Kan^R^ | Novagen |
| **b. Newly designed vectors** | | |
| pFnT7(wt) | monocistronic US fusion & expression vector; pseudo-wt; Amp^R^; lacP/Z | This study |
| pFnT7(3') | monocistronic US fusion & expression vector; 3'-StrepII-tag; Amp^R^; lacP/Z | This study |
| pFnT7(5') | monocistronic US fusion & expression vector; 5'-StrepII-tag; Amp^R^; lacP/Z | This study |
| pFcT7(wt) | monocistronic DS fusion & expression vector; pseudo-wt; Amp^R^; lacP/Z | This study |
| pFcT7(3') | monocistronic DS fusion & expression vector; 3'-StrepII-tag; Amp^R^; lacP/Z | This study |
| pFcT7(5') | monocistronic DS fusion & expression vector; 5'-StrepII-tag; Amp^R^; lacP/Z | This study |
| pAmp.RSF.3a(blue) | compatible expression vector; Amp^R^; RSF ori; lacP/Z | This study |
| pSm.CDF.3a(blue) | compatible expression vector; Sm^R^; CDF ori; lacP/Z | This study |
| **c. Selection of full expression plasmid constructs; all genes T7-controlled except pASG derivatives (Tet-controlled)** | | |
| pE_X(wt)_90/91/89/88 (**pSH4.wt**) | *hoxY, hoxH, hoxU, hoxF*; Kan^R^; ColE1 ori | This study |
| pE_X(wt)_90/91/89/88/93 (**pSH6.wt**) | *hoxY, hoxH, hoxU, hoxF, hoxI*; Kan^R^; ColE1 ori | This study |
| pE_X(wt)_90/91/89_X(5')_88 (**pSH4.Strep**) | *hoxY, hoxH, hoxU,* 5'-StrepII-*hoxF*; Kan^R^; ColE1 ori | This study |
| pE_X(wt)_90/91_X(5')_93_X(wt)_89/88 (**pSH6.Strep**) | *hoxY, hoxH,* 5'-StrepII-*hoxI, hoxU, hoxF*; Kan^R^; ColE1 ori | This study |
| pE_X(wt)_96/17/18/92/15/16/94/95 (**pE.M1**) (used in NAES4.1) | *hypF2, hypE1, hypX, hoxW, hypC1, hypD1, hypA2, hypB2*; Kan^R^; ColE1 ori | This study |
| pSm.CDF.3a_X(wt)_96/17/18/92/15/16/94/95 (**pM1**) | *hypF2, hypE1, hypX, hoxW, hypC1, hypD1, hypA2, hypB2*; Sm^R^; CDF ori | This study |
| pSm.CDF.3a_X(wt)_96/17/18/92/15/16/94/95/23 (**pM1-hoxN1**) | *hypF2, hypE1, hypX, hoxW, hypC1, hypD1, hypA2, hypB2, hoxN1*; Sm^R^; CDF ori | This study |
| pSm.CDF.3a_X(wt)_18/92/76/72/73/75/77/78 (**pM2**) | *hypX, hoxW, hypE2, hypF3, hypC2, hypD2, hypA3, hypB3*; Sm^R^; CDF ori | This study |
| pSm.CDF.3a_X(wt)_18/92/76/72/73/75/77/78/23 (**pM2-hoxN1**) | *hypX, hoxW, hypE2, hypF3, hypC2, hypD2, hypA3, hypB3, hoxN1*; Sm^R^; CDF ori | This study |
| pSm.CDF.3a_X(5')_88_X(wt)_96/17/18/92/15/16/94/95 (**pM1-*hoxF*.Strep**) (used in SHdec1) | 5'-StrepII-*hoxF, hypF2, hypE1, hypX, hoxW, hypC1, hypD1, hypA2, hypB2*; Sm^R^; CDF ori | This study |
| pSm.CDF.3a_X(wt)_89N_X(5')_88_X(wt)_96/17/18/92/15/16/94/95 (**pM1-*hoxUF*.Strep**) (used in SHdec3) | *hoxU,* 5'-StrepII-*hoxF, hypF2, hypE1, hypX, hoxW, hypC1, hypD1, hypA2, hypB2*; Sm^R^; CDF ori | This study |
| pAmp.RSF.3a_X(wt)_90/91/89/88 | *hoxY, hoxH, hoxU, hoxF*; Amp^R^; RSF ori | This study |
| pAmp.RSF.3a_X(wt)_90/91/89_X(5')_88 (**pAmp.RSF.SH4.Strep**) (used in NAES4.1) | *hoxY, hoxH, hoxU,* 5'-StrepII-*hoxF*; Amp^R^; RSF ori | This study |
| pAmp.RSF.3a_X(wt)_90/91_X(5')_93_X(wt)_89/88 | *hoxY, hoxH,* 5'-StrepII-*hoxI, hoxU, hoxF*; Amp^R^; RSF ori | This study |
| pASGwt_90_rbs3_91 (**pASGwt-*hoxYH***) (used in SHdec3) | *hoxY, hoxH* (bicistronic, one promoter); Amp^R^; ColE1 ori | This study |
| pASGwt_90_rbs3_91_rbs3_89 (**pASGwt-*hoxUYH***) (used in SHdec1) | *hoxY, hoxH, hoxU* (tricistronic, one promoter); Amp^R^; ColE1 ori | This study |
| **d. Expression plasmid constructs for deletion studies; all genes T7-controlled** | | |
| pSm.CDF.3a_X(wt)_96/17/18/92/15/16 (**pM1 ΔHypAB**) | *hypF2, hypE1, hypX, hoxW, hypC1, hypD1*; Sm^R^; CDF ori | This study |
| pSm.CDF.3a_X(wt)_96/17/18/92/94/95 (**pM1 ΔHypCD**) | *hypF2, hypE1, hypX, hoxW, hypA2, hypB2*; Sm^R^; CDF ori | This study |
| pSm.CDF.3a_X(wt)_18/92/15/16/94/95 (**pM1 ΔHypEF**) | *hypX, hoxW, hypC1, hypD1, hypA2, hypB2*; Sm^R^; CDF ori | This study |
| pSm.CDF.3a_X(wt)_18/96/17/15/16/94/95 (**pM1 ΔHoxW**) | *hypX, hypF2, hypE1, hypC1, hypD1, hypA2, hypB2*; Sm^R^; CDF ori | This study |
| pSm.CDF.3a_X(wt)_92/96/17/15/16/94/95 (**pM1 ΔHypX**) | *hoxW, hypF2, hypE1, hypC1, hypD1, hypA2, hypB2*; Sm^R^; CDF ori | This study |

The plasmid selection is restricted to the finalized expression constructs stated in the main paper. Personal nomenclature of plasmids: In plasmid names, genes are not listed by their gene names but their locus tag number (on pHG1). X(wt), X(5’) or X(3’) refer to a T7 controlled gene cassette with either a 5’- or 3’-StrepII-tag or no tag (wt). From the first gene on, a slash / in front of the gene has the same intent as the aforementioned X(..), meaning all following genes are controlled likewise. The polycistronic expression constructs relevant for this paper are two pASG derivatives with two and three structural genes, respectively. In pM1 and pM2 derivatives, a – sign followed by a protein or complex name indicates added genes to the basic constructs. Accordingly, a Δ sign indicates the protein or protein complex, whose encoding genes have been deleted from the basic constructs.
