# Supplementary material for: An Innovative Cloning Platform Enables Large-Scale Production and Maturation of an Oxygen-Tolerant [NiFe]-Hydrogenase from Cupriavidus necator in Escherichia coli
Source: PLoS One. 2013 Jul 5;8(7):e68812. doi: 10.1371/journal.pone.0068812 (PMC3702609; doi:10.1371/journal.pone.0068812)
Supplement: Table S3 — Recombinant E. coli strains and activities for maturation studies (deletion and substitution). (DOCX) [file pone.0068812.s008.docx]

**Table S3.** Recombinant *E. coli* strains and activities for maturation studies (deletion and substitution).

| **Category** | **Strain^a^** | **Specific activity [U·mg^-1^]^b^** | **Normalized** |
| --- | --- | --- | --- |
| **Control strains** | *E. coli* BL21Star(DE3)  K0 [K1A ΔM1] | **< 0.001**  **< 0.001** |  |
| **M1 deletion strains** | K1A [pSH4.wt + **pM1**] | **1.953** ± 0.236 | **100%** |
|  | K1A ΔHypA2B2 | **0.188** ± 0.026 | **9.6%** |
|  | K1A ΔHypC1D1 | **0.030** ± 0.003 | **1.6%** |
|  | K1A ΔHypE1F2 | **0.024** ± 0.004 | **1.2%** |
|  | K1A ΔHoxW | **< 0.001** | **0%** |
|  | K1A ΔHypX | **1.498** ± 0.245 | **77%** |
| **M2 strain** | K1B [pSH4.wt + **pM2**] | **0.15** ± 0.018 | **7.7 %** |

^a^ All recombinant strains were generated from *E. coli* BL21Star™ (DE3).

^b^ Specific activities were determined in extracts from cells obtained in three independent ‘autoinduction’ batches. Growth conditions in these experiments were as described in the methods section, except the optimized conditions for the preparatory cultures were not yet developed. Hence, inoculation was simply carried out by 1:50 (v/v) dilution of an overnight culture with the induction medium. Given values are arithmetic means of the triplicate measurements. Statistical values indicated (±) represent standard deviations. Normalization was calculated based on the arithmetic mean values. 1 Unit is defined as the H_2_-mediated reduction of 1 µmol NAD^+^ per minute.
