# Supplementary material for: An Innovative Cloning Platform Enables Large-Scale Production and Maturation of an Oxygen-Tolerant [NiFe]-Hydrogenase from Cupriavidus necator in Escherichia coli
Source: PLoS One. 2013 Jul 5;8(7):e68812. doi: 10.1371/journal.pone.0068812 (PMC3702609; doi:10.1371/journal.pone.0068812)
Supplement: Table S4 — Selection of three independent purification trials for determination of the ideal buffer composition for SH stabilization in E. coli extracts. In each case, 1 gram of wet-packed cells (strain SH1F; four-subunit SH variant 1) from the same ‘autoinduction’ batch was used for purification. (DOCX) [file pone.0068812.s009.docx]

**Table S4.** Selection of three independent purification trials for determination of the ideal buffer composition for SH stabilization in *E. coli* extracts. In each case, 1 gram of wet-packed cells (strain SH1F; four-subunit SH variant 1) from the same ‘autoinduction’ batch was used for purification.

| **Specification of purification conditions^a^** | **CFE specific activity [U·mg^-1^]** | **Final specific activity [U·mg^-1^]^b^** | **Purification fold** | **Yield^b^** |
| --- | --- | --- | --- | --- |
| (1) Opening buffer air saturated | 0.6 | 36.2 | 61 | 16% |
| (2) Opening buffer saturated with pure O_2_ | 0.59 | 51.7 | 88 | 16% |
| (3) Opening buffer with 50 mM succinate, suspension under Argon atmosphere | 0.69 | 125.5 | 181 | 19% |

^a^ Methodology: Cells were opened in 5 mL of the specified buffer supplemented with DNase, 0.05 mM PMSF and 25 µg avidin. Following cell opening by five consecutive cycles of freeze-thaw, the extracts were cleared by ultracentrifugation (45 min; 140.000 *g*; 2 °C). During the initial steps in (3), the Argon atmosphere was maintained. Cell-free extracts were loaded onto a 1 mL StrepTactin Superflow^®^ gravity flow column and purified according to the manufacturer’s instructions. SH-containing fractions were pooled, concentrated and loaded onto a preequilibrated Superdex 200 HR 10/300 gel filtration column for polishing.

^b^ Final specific activity refers to the SH H_2_:NAD^+^ physiological activity, measured under anaerobic conditions after the gel filtration step. The yield refers to the residual activity captured after the gel filtration step in comparison to the initial CFE activity. 1 Unit is defined as the H_2_-mediated reduction of 1 µmol NAD^+^ per minute.
