# Supplementary material for: An Innovative Cloning Platform Enables Large-Scale Production and Maturation of an Oxygen-Tolerant [NiFe]-Hydrogenase from Cupriavidus necator in Escherichia coli
Source: PLoS One. 2013 Jul 5;8(7):e68812. doi: 10.1371/journal.pone.0068812 (PMC3702609; doi:10.1371/journal.pone.0068812)
Supplement: Table S5 — Properties of the native and recombinant SH variants purified in this study. (DOCX) [file pone.0068812.s010.docx]

**Table S5.** Properties of the native and recombinant SH variants purified in this study.

| **Property^a^** | **SH_wt_ (*Cn*)^b^** | **SH_tet, var1_ (*Ec* SH1F)^b^** | **SH_hex, var2_ (*Ec* SH2F)^b^** |
| --- | --- | --- | --- |
| Stability on ice, CFE; air saturated KPi buffer | t_1/2_ = 12 h | t_1/2_ = 1,1 h | |
| Stability on ice, CFE; KPi buffer + 50 mM succinate under an Argon atmosphere | t_1/2_ = 24 h | t_1/2_ = 3,5 h | |
| Stability -20 °C, purified | 6 months; >80% | 6 months; >75% | 6 months; >75% |
| Stability -80 °C, purified | 6 months; >90% | | |
| Stability under H_2_/N_2_, purified | activity lost within 6 hours, and within 40 min in the presence of NADH (on ice) | | |
| Hypothetical enzyme stoichiometric composition | 6 Subunits (HoxFUYHI_2_) | 4 Subunits (HoxUYH, 5'-StrepII-tagged HoxF) | 6 Subunits (HoxFUYH, 5'-StrepII-tagged HoxI_2_) |
| Iron content [mol/mol enzyme] | 16-19 | 15-17 | 15-18 |
| Molecular weight (gel filtration) | not determined^c^ | 171 kDa | 213 kDa |
| Aerobic specific activity of purified fraction^d^ | 101 U·mg^-1^ | 152 U·mg^-1^ | 173 U·mg^-1^ |
| Anaerobic specific activity of purified fraction^d^ | 130 U·mg^-1^ | 192 U·mg^-1^ | 227 U·mg^-1^ |

^a^ Stabilities in cell-free extracts (CFE) were determined by activity assays every 30 min (recombinant variants) or 120 min (wildtype variant). Extract activities were similar to the ones obtained at purification start (see Tables 3-5). Values are arithmetic means from three independent extract preparations.

^b^ The preparations listed in Tables 3-5 were used for determination of individual properties.

^c^ The molecular weight of the native SH has been determined in a previous study to about 205 kDa [Schneider K, Schlegel HG (1976) Purification and properties of soluble hydrogenase from *Alcaligenes eutrophus* H 16. Biochim Biophys Acta 452: 66-80].

^d^ The activity under aerobic assay conditions is 20-25% reduced compared to anaerobic activity measurements. This is in line with the data presented by [Bleijlevens B, Buhrke T, van der Linden E, Friedrich B, Albracht SP (2004) The auxiliary protein HypX provides oxygen tolerance to the soluble [NiFe]-hydrogenase of Ralstonia eutropha H16 by way of a cyanide ligand to nickel. J Biol Chem 279: 46686-46691] and [van der Linden E, Burgdorf T, de Lacey AL, Buhrke T, Scholte M, et al. (2006) An improved purification procedure for the soluble [NiFe]-hydrogenase of Ralstonia eutropha: new insights into its (in)stability and spectroscopic properties. J Biol Inorg Chem 11: 247-260]. These groups provided a profound interpretation of this phenomenon in the abovementioned papers. 1 Unit is defined as the H_2_-mediated reduction of 1 µmol NAD^+^ per minute.
