# Supplementary material for: An Innovative Cloning Platform Enables Large-Scale Production and Maturation of an Oxygen-Tolerant [NiFe]-Hydrogenase from Cupriavidus necator in Escherichia coli
Source: PLoS One. 2013 Jul 5;8(7):e68812. doi: 10.1371/journal.pone.0068812 (PMC3702609; doi:10.1371/journal.pone.0068812)
Supplement: Table S6 — Activation behavior and kinetic properties of native and recombinant SH preparations upon application of different assay conditions. (DOCX) [file pone.0068812.s011.docx]

**Table S6.** Activation behavior and kinetic properties of native and recombinant SH preparations upon application of different assay conditions.

| **SH variant** | **Sample^a^** | **Amount^b^** | **Activation** | **Assay^c^** | **lag time [sec]^d^** | **v_max_ [U·mg^-1^]^e^** |
| --- | --- | --- | --- | --- | --- | --- |
| SH_wt_ (*Cn*) | Early preparation | 46 nM | 5 µM NADH | Anaerobic; 5 mM NAD^+^; 30 µM H_2_ | n. d. | 69,3 |
|  |  |  | 40 µM NADPH |  | n. d. | 68 |
|  |  |  | no |  | 145 | 62,9 |
|  |  |  | 5 µM NADH | Aerobic; 5 mM NAD^+^; 30 µM H_2_ | n. d. | 56,4 |
| SH_tet, v1_ (rec) | Preparation 1 | 49 nM | 5 µM NADH | Anaerobic; 5 mM NAD^+^; 30 µM H_2_ | n. d. | 51,5 |
|  |  |  | 40 µM NADPH |  | 185 | 49,2 |
|  |  |  | no |  | 195 | 48,7 |
|  |  |  | 5 µM NADH | Aerobic; 5 mM NAD^+^; 30 µM H_2_ | n. d. | 40,9 |
|  | Preparation 2 | 35 nM | 5 µM NADH | Anaerobic; 5 mM NAD^+^; 30 µM H_2_ | n. d. | 84,2 |
|  |  |  | 40 µM NADPH |  | 95 | 80,8 |
|  |  |  | no |  | 95 | 80 |
|  |  |  | 5 µM NADH | Aerobic; 5 mM NAD^+^; 30 µM H_2_ | n. d. | 62,1 |
|  |  |  | no |  | 190 | 60,3 |
| SH_hex, v2_ (rec) | Preparation 1 | 55 nM | 5 µM NADH | Anaerobic; 5 mM NAD^+^; 30 µM H_2_ | n. d. | 43,5 |
|  |  |  | 40 µM NADPH |  | n. d. | 43,8 |
|  |  |  | no |  | 185 | 41,9 |
|  |  |  | 5 µM NADH | Aerobic; 5 mM NAD^+^; 30 µM H_2_ | n. d. | 36,5 |
|  | Preparation 2 | 37 nM | 5 µM NADH | Anaerobic; 5 mM NAD^+^; 30 µM H_2_ | n. d. | 79,5 |
|  |  |  | 40 µM NADPH |  | n. d. | 79,2 |
|  |  |  | no |  | 90 | 79,2 |
|  |  |  | 5 µM NADH | Aerobic; 5 mM NAD^+^; 30 µM H_2_ | n. d. | 62,2 |
|  |  |  | no |  | 175 | 59,9 |
|  | Cell-free extract; strain SH2F | 20 µL of diluted CFE (1:50 v:v) | 5 µM NADH | Anaerobic; 5 mM NAD^+^; 30 µM H_2_ | n. d. | 0,99 |
|  |  |  | 40 µM NADPH |  | n. d. | 1,05 |
|  |  |  | no |  | n. d. | 0,99 |
|  |  |  | 5 µM NADH | Aerobic; 5 mM NAD^+^; 30 µM H_2_ | n. d. | 0,88 |
|  |  |  | no |  | 20 | 0,82 |
| SH_tet_ ∆HypX | Cell-free extract; strain K1A ∆HypX | 20 µL of diluted CFE (1:25 v:v) | 5 µM NADH | Anaerobic; 5 mM NAD^+^; 30 µM H_2_ | n. d. | 0,83 |
|  |  |  | 40 µM NADPH |  | n. d. | 0,83 |
|  |  |  | no |  | n. d. | 0,79 |
|  |  |  | 5 µM NADH | Aerobic; 5 mM NAD^+^; 30 µM H_2_ | n. d. | 0,75 |
|  |  |  | no |  | 65 | 0,16^f^ |

^a^ For activation and kinetic studies, different preparations of the three SH variants were tested. Preparation 1 of the recombinant variants stemmed from early purification trials. The quality of the SH preparation always has a substantial impact on the self-activation behavior. Preparation 2, which represents purified SH variants of higher quality, was used for comparison and for aerobic measurements. Self-activation is a phenomenon, which is faced when no activator is added to the assay. A small portion of active enzyme molecules catalyses H_2_-mediated NAD^+^ reduction, and the product, NADH, then activates the inactive hydrogenase molecules. The time of self-activation correlates with the “lag time”, that is, the time from the assay start to the point, where maximum velocity is achieved. In order to compare the activation and aerobic behavior of the ∆HypX SH variant, cell-free extracts from the respective expression strain (K1A ∆HypX) were used. To allow a direct comparison of this O_2_-sensitive variant with the HypX^+^-variants, an extract of SH_variant2_ was subjected to the same assays.

^b^ As outlined in the methods section of the main paper, we always used 20 µL of enzyme sample in 1 mL assay. For the purified preparations, we used proper dilutions (final amount is given in this table), while cell-free extracts were routinely used in a 1:50 or 1:25 (v/v) diluted form.

^c^ Aerobic and anaerobic measurements were performed as outlined in the methods section of the main paper.

^d^ n. d. = not determined (no lag phase); The lag phase was determined as the time between the assay start and the point, where maximum velocity was achieved. Cell-free extracts normally showed no lag phase in the absence of an activator, probably due to a significant amount of reduced pyridine nucleotides present in the cellular environment (and, thus, in the extract). However, in aerobic assays without additional provision of activators, a small lag phase is normally observed even with CFE samples.

^e^ 1 Unit is defined as the H_2_-mediated reduction of 1 µmol NAD^+^ per minute.

^f^ The ∆HypX SH variant is unstable towards O_2_. In aerobic assays, the enzyme shows normal behavior when an activator is present. However, in an activator-free aerobic assay following a significant lag phase, maximum velocity is only observed for about 10-20 seconds (and only to about 20% of the maximum activity achieved in anaerobic assays), before the absorption line remains constant (no further activity). These results are in line with the data provided by [Bleijlevens B, Buhrke T, van der Linden E, Friedrich B, Albracht SP (2004) The auxiliary protein HypX provides oxygen tolerance to the soluble [NiFe]-hydrogenase of *Ralstonia eutropha* H16 by way of a cyanide ligand to nickel. J Biol Chem 279: 46686-46691.].
